# Pairwise comparative analysis of six haplotype assembly methods based on users’ experience

**DOI:** 10.1186/s12863-023-01134-5

**Published:** 2023-06-29

**Authors:** Shuying Sun, Flora Cheng, Daphne Han, Sarah Wei, Alice Zhong, Sherwin Massoudian, Alison B. Johnson

**Affiliations:** 1 Department of Mathematics, Texas State University, San Marcos, TX USA; 2 Carnegie Mellon University, Pittsburgh, PA USA; 3 Massachusetts Institute of Technology, Cambridge, MA USA; 4 Clements High School, Sugar Land, TX USA; 5 Texas State University, San Marcos, TX USA; 6 Texas A & M University, College Station, TX USA

## Abstract

**Background:**

A haplotype is a set of DNA variants inherited together from one parent or chromosome. Haplotype information is useful for studying genetic variation and disease association. Haplotype assembly (HA) is a process of obtaining haplotypes using DNA sequencing data. Currently, there are many HA methods with their own strengths and weaknesses. This study focused on comparing six HA methods or algorithms: HapCUT2, MixSIH, PEATH, WhatsHap, SDhaP, and MAtCHap using two NA12878 datasets named hg19 and hg38. The 6 HA algorithms were run on chromosome 10 of these two datasets, each with 3 filtering levels based on sequencing depth (DP1, DP15, and DP30). Their outputs were then compared.

**Result:**

Run time (CPU time) was compared to assess the efficiency of 6 HA methods. HapCUT2 was the fastest HA for 6 datasets, with run time consistently under 2 min. In addition, WhatsHap was relatively fast, and its run time was 21 min or less for all 6 datasets. The other 4 HA algorithms’ run time varied across different datasets and coverage levels. To assess their accuracy, pairwise comparisons were conducted for each pair of the six packages by generating their disagreement rates for both haplotype blocks and Single Nucleotide Variants (SNVs). The authors also compared them using switch distance (error), i.e., the number of positions where two chromosomes of a certain phase must be switched to match with the known haplotype. HapCUT2, PEATH, MixSIH, and MAtCHap generated output files with similar numbers of blocks and SNVs, and they had relatively similar performance. WhatsHap generated a much larger number of SNVs in the hg19 DP1 output, which caused it to have high disagreement percentages with other methods. However, for the hg38 data, WhatsHap had similar performance as the other 4 algorithms, except SDhaP. The comparison analysis showed that SDhaP had a much larger disagreement rate when it was compared with the other algorithms in all 6 datasets.

**Conclusion:**

The comparative analysis is important because each algorithm is different. The findings of this study provide a deeper understanding of the performance of currently available HA algorithms and useful input for other users.

**Supplementary Information:**

The online version contains supplementary material available at 10.1186/s12863-023-01134-5.

## Introduction

**Fig. 1:**
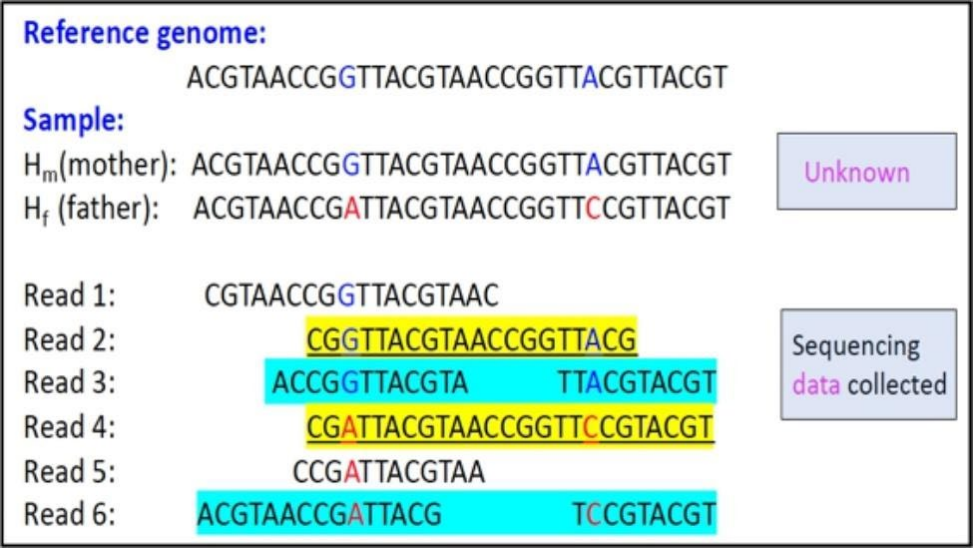
Example of haplotype assembly using DNA sequencing reads

Haplotype information can be used to study a population and generate markers and maps as a means of understanding how genetic variation evolves and contributes to phenotypes. Certain haplotypes may be associated with diseases or traits in a population [1, 2]. With the constant development of new DNA sequencing technologies in the past two decades, it has become possible to reconstruct haplotypes for genetic studies using sequencing data. The reconstruction of haplotypes from DNA sequencing reads is called haplotype assembly (HA). Figure 1 showed a hypothetical example of haplotype assembly, that is, reconstructing haplotypes of two single nucleotide variants SNVs (or positions) using DNA sequencing reads. In this figure, there were 6 different DNA sequencing reads. Sequencing reads 1 and 5 only covered one SNV, so they could not be used directly to identify haplotypes. Reads 2 and 4 were single-end reads that were long enough to cover two SNVs and could be used to infer unknown haplotypes. Reads 3 and 6 were paired-end reads that covered two SNVs, so they could be used to infer haplotypes as well.

HA is important in order to understand and interpret genetic variants, as well as these variants’ association with diseases in certain groups of individuals [3]. Therefore, there have been numerous HA methods developed over the last 10 to 15 years as mentioned in recent review papers [4–7]. These methods used different approaches. Some of them (e.g., HapCUT2, MixSIH, PEATH, and ProbHap) were developed based on probability models (i.e., they were probability-based) [1, 3, 8–20], whereas some (e.g., WhatsHap and SDhaP, and GenHap) focused on addressing a minimum error correction (MEC) problem [21–23]. With the development of these methods and their own unique features, the precision of haplotype assembly has steadily improved over the years. However, there were still challenges in haplotype assembly for different users and researchers who tried to develop new HA methods. First, because each algorithm had its own advantages and disadvantages, users might not know which one to choose. Second, users might find it difficult to figure out how to get an HA algorithm to run properly. Third, users might not understand input and output files easily due to the lack of good user manuals. Finally, most methods were only compared with 2 or 3 other algorithms, and few studies compared a wide range of HA methods. Because of the above challenges, users might not fully understand the algorithms they used, as well as the advantages and disadvantages of their respective performances. Therefore, it is important to compare these algorithms to help users know the HA problem better. A comparison study can also help other researchers to understand this topic, and thus develop more accurate and efficient HA algorithms.

In this paper, the authors compared 6 HA methods (or software packages): HapCUT2 [1], MixSIH [14], PEATH [16], WhatsHap [22], SDhaP [21], and MAtCHap [24]. Table 1 showed the web pages, languages used by the 6 HA methods, and software versions (or installation dates). One commonly used programming language amongst these 6 HA algorithms was C + + or C. MixSIH also used Ruby in addition to C++, whereas WhatsHap and HapCUT2 used Python. Different from other HA algorithms, MAtCHap used R and Perl. HapCUT2, WhatsHap, and SDhaP also relied on additional software in order to successfully complete haplotype assembly. HapCUT2 required extractHAIRS (Extract HAplotype Informative Reads) and htslib. WhatsHap relied on the Conda package. SDhaP required the installation of ATLAS and LAPACK. Further detailed information about these HA methods can be found in their webpages. In addition, these 6 methods were the representatives of the following three approaches: using a probability model (HapCUT2, MixSIH, and PEATH), addressing a MEC problem (WhatsHap and SDhaP), and solving a maximization problem (MAtCHap). More detailed methodological and analytical comparisons were summarized in later sections. The 6 HA methods were compared using the run time (i.e., CPU time), haplotype block, SNV number per block, and switch distance (or error) rate. Datasets, comparison methods, and findings were shown in different sections.

**Table 1:** Webpages and languages used by 6 HA algorithms

Method	Language	Webpage	Version (or Date)
HapCUT2	C, Python	https://github.com/vibansal/HapCUT2	07/2020 installed
MixSIH	C++, Ruby	https://github.com/hmatsu1226/MixSIH https://sites.google.com/site/hmatsu1226/software/mixsih	Version 1.0.0
PEATH	C++	https://github.com/jcna99/PEATH	07/2020 installed
WhatsHap	C++, Python	https://WhatsHap.readthedocs.io/en/latest/index.html	Version 0.18
SDhaP	C	https://sourceforge.net/projects/SDhaP/files/	07/2020 installed
MAtCHap	R, Perl	https://sourceforge.net/projects/MAtCHap/.	Version 1.0

The columns (left to right) represent haplotype assembly method, programming language(s), webpages, and versions (or installation dates)

## Comparison method

This section included the description of data to be used, the workflow of 6 HA algorithms, a pairwise comparison analysis method (i.e., the analytical part), as well as a methodological comparison of the 6 HA algorithms (i.e., the conceptual part). Note, the methodological comparison of the 6 HA algorithms was also a part of the comparison results, which was supposed to be included in the Results section. However, since this part did not involve any analytical results regarding each HA algorithm’s performance, the authors included them in this section. Next, analysis datasets would be described, which included the publicly available sequencing data and known haplotypes.

### DNA sequencing data and “gold standard” (or known) haplotypes of NA12878

To compare the 6 HA methods, DNA sequencing datasets of the sample named NA12878 were used in this study. NA12878’s genome was commonly used to study haplotype assembly in previous research. NA12878 was a female Utah resident with Northern and Western European ancestry [25] and a sample (HG001) from the 1000 Genomes Project. The NA12878 sample has been sequenced many times using a variety of sequencing technologies [25–27]. Different datasets have become available for this sample, which included integrated variant call sets, exome, low coverage whole genome sequencing (WGS), PCR-free high coverage sequencing, HD genotype chip, and targeted exons. The technologies used for sequencing NA12878 included Hi-C [28], fosmid-based [29], 10X genomics linked-read [30], PacBio SMRT [31], 454-sequencing, and Illumina HiSeq 2500 [32].

For the sake of convenience, two versions of the aligned sequencing data were used. These two datasets were publicly available in the Binary Alignment Map (BAM) format and were called ENA-hg19 and ENA-hg38 data. ENA-hg19 and ENA-hg38 mean that the aligned datasets were downloaded from the European Nucleotide Archive (ENA) [27], and the sequencing reads were aligned to the hg19 and hg38 reference genome respectively. The ENA-hg19 dataset included 2 × 100 bp paired-end reads generated using the Illumina HiSeq 2000 with > 30X coverage. The ENA-hg38 dataset included 2 × 150 bp paired-end reads generated using the Illumina NovaSeq 6000 with > 30X coverage. More detailed information can be found at the ENA web page [27]. The ftp sites of these datasets have been listed in the Availability of Data and Materials section. For the sake of simplification, they were called hg19 and hg38 data in the later sections.

The hg38 version of NA12878 true (or known) haplotype dataset was downloaded from the International Genome Sample Resource (IGSR) [26]. The ftp site of this dataset was listed in the Availability of Data and Materials section. To the best of the authors’ knowledge, this dataset can be used as a “gold standard” (or a set of “high confidence” haplotypes of NA12878) to compare different HA algorithms. To simplify the analysis, the authors only used chromosome 10 (chr10) sequencing data. For chr10, based on the hg38 reference genome, there were 133 million (133,797,422) positions. Out of the total 133 million positions, there were only 3.6 million (3,632,297) SNV positions in the hg38 version known haplotypes. Among these 3.6 million potential SNV positions, 3.4 million (3,455,455) positions were homozygous reference alleles (i.e., “0|0”), and the other positions were alternative allele homozygous sites (67,126 “1|1” positions) or heterozygous sites (53,462 “0|1” and 56,354 “1|0” positions). For all the 3.6 million (3,632,297) SNV positions, 3,591,460 positions were lifted over or converted to the hg19 reference genome. For this conversion, the following three types of positions were removed: positions that could not be converted to hg19, positions that were mapped to other chromosomes but not chr10, and positions that were mapped to more than one place on hg19 chr10. These 3.6 million (3,632,297) hg38 SNV positions and 3.5 million (3,591,460) hg19 SNV positions were used for further analysis later. Note, in the NA12878 “gold standard” haplotype dataset, all positions were likely to be single nucleotide polymorphisms (SNPs) as they were typically inferred as “high confidence” positions. However, the authors still called these positions SNVs in case that some of them might not be validated SNPs yet. For the rest of this paper, the term SNV was used as it was still unknown whether the variants inferred by each HA algorithm were SNPs or not.

The two datasets, hg19 and hg38, and the known haplotype data (or “gold standard”) described above were used in order to compare the 6 HA methods. To study the impact of sequencing depth (DP) or coverage, the input datasets were filtered based on 3 levels: DP1, DP15, and DP30. The specified number after the term DP was the number of reads that cover each position or base (i.e., ≥ 1X, ≥ 15X, and ≥ 30X). The number of DNA fragments in each dataset was shown in Table 2. This table shows that as the filtering level increases from DP1 to DP30, the DNA fragment number decreases significantly because low-coverage or low-quality sequencing reads were removed.

**Table 2:** Numbers of DNA fragments of hg19 and hg38 data based on 3 depth levels

	hg19	hg38
DP1	966,935	787,932
DP15	924,996	785,698
DP30	451,625	570,691

Note that, a SNP or SNV with just 1X coverage was not a valid or meaningful calling. By setting DP1 (1X), the purpose was not to identify a specific SNP (or SNV) or haplotype with just 1X. Instead, it was to see how well different algorithms would perform and how well they agreed with each other when users did not do any filtering based on coverage. Users might not know what coverage level should be used to do the filtering, and whenever there was a filtering, some SNPs (SNVs) could be filtered out. Therefore, all algorithms were compared with DP1 (1X), DP15 (15X), and DP30 (30X) to show users what they might expect if they analyzed a new dataset by themselves.

### Workflow of 6 HA methods

**Fig. 2:**
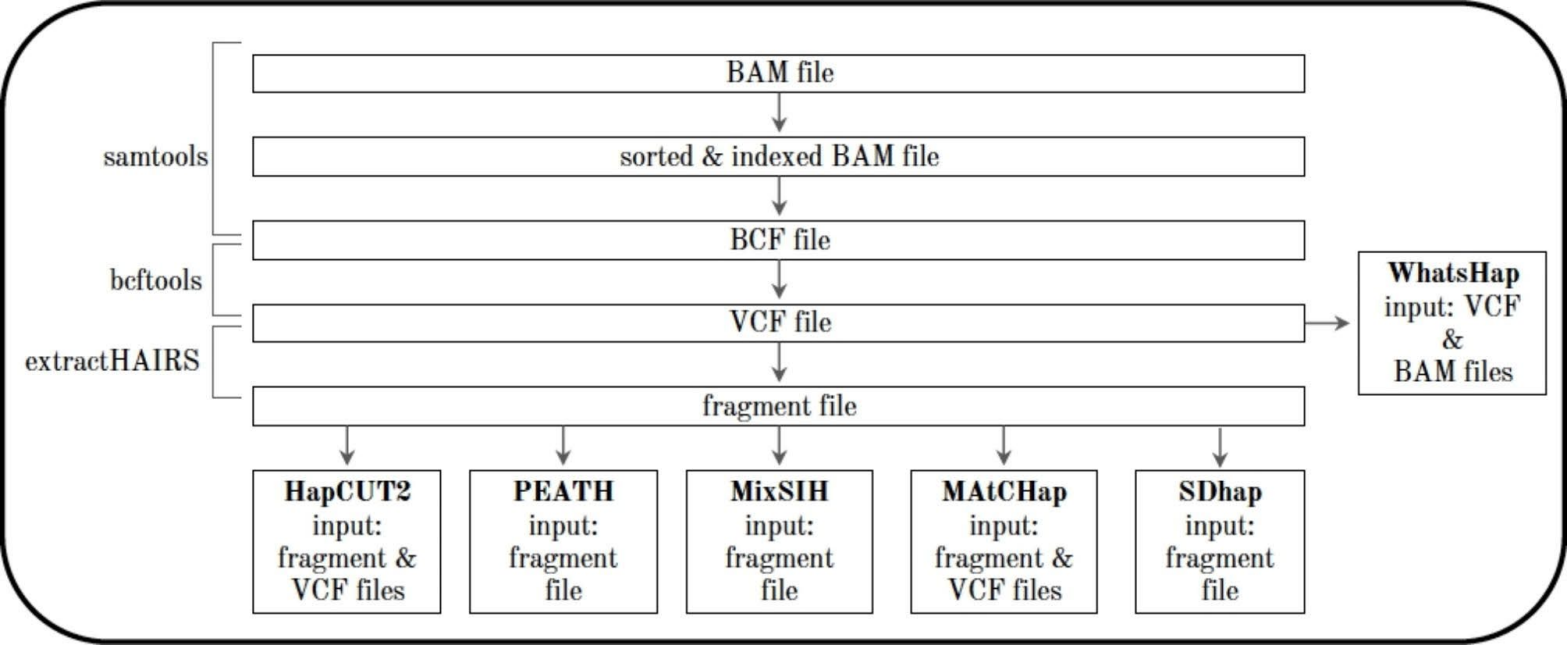
The workflow of 6 HA algorithms. This workflow is about converting BAM files to generate the required input format for each software package

Figure 2 showed the workflow of 6 HA methods (or algorithms). This workflow was summarized based on the authors’ experience of using these HA methods. It began with the NA12878 BAM file, which was sorted using the samtools. The chr10 was then extracted from the BAM file to create a sorted SAM file, which was then converted back to a sorted BAM file, and finally to a BIM Collaboration Format (BCF) file. Next, bcftools were used to convert a BCF file to a VCF file. Since WhatsHap only required a VCF file and a BAM file (meaning it does not use a DNA sequencing fragment file), it can be run right after the VCF file was generated. For the other HA algorithms, a fragment file was required. By using the tool extractHAIRS (a script provided on HapCUT2 web page), a fragment file could be generated from the VCF file, which allowed HapCUT2, MixSIH, PEATH, MAtCHap, and SDhaP to have the required input format to run.

The authors used DNA sequencing data of the sample NA12878, that is, the hg19 and hg38 data, and then filtered them based on 3 different sequencing depth levels (DP1, DP15, and DP30). They ran the six HA software packages, HapCUT2, MixSIH, PEATH, WhatsHap, SDhaP, and MAtCHap. The original output files were then reformatted, using Perl and R scripts, to obtain the SNV and block summary values. These values included the number of SNVs, the number of haplotype blocks, mean number of SNVs per block, as well as the maximum, minimum, and quartiles for the number of SNVs per block. The results between the various runs were then compared to find differences or disagreements.

### Pairwise comparison analysis method (i.e., analytical comparison)

The key purpose of this study was to conduct comparative analysis. Before any pairwise comparisons, the authors used a “gold standard” (i.e., a set of known haplotypes) for both hg19 and hg38 datasets that were publicly available. The analysis was done by cross comparing the haplotype output file from each HA with the so-called “gold standard” haplotype file. This comparison was done to see how many SNVs inferred by each HA overlapped with the positions in the “gold standard”. The authors first showed the non-pairwise comparison result and then explained why they did the pairwise comparison in the Results section.

After running the 6 HA algorithms on 6 datasets (hg19 and hg38, each with 3 filtering levels: DP1, DP15, and DP30), the raw output files were obtained, see examples of raw output files in the Table 1 in the Supplementary Material 1. HapCUT2, MixSIH, PEATH, and SDhaP had a similar output format. The first column in this supplemental table was the SNV index in the input VCF file. The second and third columns were the haplotypes on two chromosomes. The other columns were detailed explanations. The MAtCHap and WhatsHap raw outputs were VCF files. These output files were then compared using run time, haplotype block, SNV, and switch error.

The authors did pairwise comparison for haplotype blocks, SNVs, and switch error rates by performing analysis on each pair of HA methods. That is, one HA was used as a reference or standard (called HA1), and the secondary HA (called HA2) was compared to the HA1. This pairwise comparison was done block by block as shown in Table 3. An agreement was reported if the number of SNVs in a particular block matched, and if it did, the comparison algorithm then checked if the alleles matched or not (see the 5th column, “hap.match”). In the end, each comparison generated both block and SNV disagreement counts, which were converted to percentages to compare various HA algorithms that had different blocks and SNVs.

For switch errors, the authors compared each pair of HA algorithms and used one as the standard or reference (i.e., HA1). They checked two haplotypes block by block and reported the number of switches needed to make the alleles of the secondary HA (i.e., HA2) match the reference HA’s alleles (see the last column of Table 3). The comparison function ultimately reported the total number of switches, as well as other statistics such as the blocks with no switches, with switches, and with no comparisons due to different numbers of SNVs. In order to compare based on the switch distance metric thoroughly, the authors zoomed in to compare all HA methods from different perspectives by defining the following 12 specific metrics.

1. blk.w.0sw: Total number of blocks (blks) that 2 HAs agreed with each other, i.e., no switch (sw = 0).
2. blk.w.NAsw: Total number of blocks whose SNV numbers did not match (sw = “NA”). That is, the switch distance could not be checked.
3. blk.w.sw: Total number of disagreement blocks with switch counted (sw > 0).
4. snv.in.blk.w.0sw: Total number of SNVs in blocks with 0 switches (sw = 0).
5. snv.in.blk.w.NAsw: Total number of SNVs in blocks with NA switches (sw = “NA”).
6. snv.in.blk.w.sw: Total number of SNVs in blocks with switches (sw > 0).
7. snv.per.blk.w.0sw: Average number of SNVs in blocks with 0 switches (sw = 0).
8. snv.per.blk.w.NAsw: Average number of SNVs in blocks with NA switches (sw= “NA”).
9. snv.per.blk.w.sw: Average number of SNVs in blocks with switches (sw > 0).
10. total.sw: Total number of switches for all disagreement blocks with an equal number of SNVs in 2 HA methods.
11. snv.by.sw: snv/sw, it was for blocks with sw > 0 (not for ALL disagreement blocks).
12. sw.per.blk: sw/blk, it was for blocks with sw > 0 (not for ALL disagreement blocks).

**Table 3:** A simple example of comparing two HA algorithms

block.ID	HA1.SNV.num	HA2.SNV.num	SNV.match	hap.match	switch.count
1	2	1	-	-	NA
2	7	4	-	-	NA
3	4	4	match	-	1
4	23	9	-	-	NA
5	2	2	match	match	0
6	2	2	match	match	0

The columns are block ID, number of SNVs in HA1, number of SNVs in HA2, SNV match flag, haplotype match flag, and switch count. “-” means SNV numbers or haplotypes of HA1 and HA2 do not match. In the switch count column, NA means no need to check switch distance because those HA1 and HA2’s SNV numbers do not match. The number (e.g., 0 or 1) in the last column means the number of switches needed

### Methodological comparison (i.e., the conceptual comparison)

Below is the methodological or conceptual comparison of the 6 HA methods based on the following aspects: models and features, input files, and comparison metrics.

#### Models and features

The 6 HA methods utilized a variety of statistical models or algorithms as shown in Table 4. HapCUT2 used a maximum likelihood-based model. It was designed to work across a wide array of sequencing technologies [1]. MixSIH used a probabilistic mixture model where each fragment was emitted independently of other fragments [14]. PEATH utilized a probabilistic evolutionary algorithm that used a fitness function to identify candidates for optimization [16]. WhatsHap and SDhaP focused on addressing the MEC problem. WhatsHap considered HA as a weighted minimum error correction (wMEC) problem with read coverage as the only fixed parameter. It solved the wMEC problem using dynamic programming to find a partition of DNA sequencing reads [22]. SDhaP attempted to find the optimal solution for the MEC problem [21]. It approached HA as a correlation-clustering problem and aimed to solve low-rank semidefinite programming optimization problems. MAtCHap’s model was based on the maximum allele co-occurrence. It aimed to reconstruct haplotype structures of all coverages with a high accuracy [24].

Assumptions were often made to ensure the modeling environment was under control and stable. HapCUT2 assumed that the read fragments were independent and the heterozygous sites were known in advance [1]. MixSIH assumed that the sequence error rate was not dependent on fragments or positions and mixture probabilities were equal [14]. The PEATH method assumed that all input variables were independent. WhatsHap assumed that the allele with the higher alignment score was supported by sequencing reads [22]. It also assumed that recombination events had the same chance to occur at any given position [22].

The 6 HA methods often considered different features in their models. The following features were commonly used by at least 2 of the 6 HA methods as shown in Table 4: Sequencing Error (Seq Error), Sequencing Coverage (Seq Cov), Sequencing Read Length (Read Len), and Objective Function (Obj Fun). Below is the summary for each of them. Sequencing errors are mistakes of reading specific bases due to the limitations of sequencing technologies. PEATH and SDhaP considered sequencing errors in their models. PEATH attempted to identify the minimal sum of the quality-weighted errors from two haplotypes, and this was done using the Phred quality scores and probability of sequencing errors [16]. Sequencing coverage is the number of reads covering a specific base. Five methods, except PEATH, incorporated sequencing coverage in their models. HapCUT2’s switch error rates decreased as sequencing coverage increased [1], and this appeared to be the case with MAtCHap, as the switch + mismatch error rate decreased as the coverage increased [24]. MAtCHap also concluded that its run time generally increased as coverage increased, although the change in run time was not as significant as that of other algorithms [24]. Read length is the number of bases sequenced from a DNA fragment. 3 papers had read length as an evaluation method: HapCUT2, WhatsHap, and MixSIH. The HapCUT2 paper showed that the run time was dependent on the read length [1]. However, WhatsHap showed that the run time was not affected by the read lengths, which made it more suitable for larger sequencing datasets [22].

**Table 4:** Models, assumptions, and features of 6 HA algorithms

Method	Model or algorithms	Assumptions	Seq Error	Seq Cov	Read Len	Obj Fun
HapCUT2	maximum likelihood-based	Heterozygous sites known in advance; allele count independent		X	X	X
MixSIH	probabilistic mixture model	sequence error rate independent of fragments and positions; mixture probabilities are equal (pm(0) = pm(1) = 0.5 )		X	X	X
PEATH	probabilistic evolutionary algorithm	All input variables are independent, no copy number variation	X			X
WhatsHap	Fixed-parameter tractable algorithm, dynamic programming	Allele with higher alignment score is assumed to be supported by the read, variants are sorted by position, recombination events equally likely at any position		X	X	X
SDhaP	MEC		X	X		X
MAtCHap	maximum allele co-occurrence			X		X

The second column is the model or algorithm used by each HA method. The third column is the assumption used by each HA algorithm. The fourth to seventh columns are features or factors that were considered by the authors of each HA method. These features/factors are sequencing error (Seq Error), sequencing coverage or depth (Seq Cov), sequencing read length (Read Len), and objective function (Obj Fun). “X” in each column means that the HA method in that row incorporates that feature or metric. Some cells in the assumption column are left blank, which means that no clear assumptions can be found in the corresponding papers.

HA algorithms often use different objective functions. One of the most common objective functions was the likelihood function, which was implemented in the MixSIH and HapCUT2 methods [1, 14]. Another common objective function was the MEC, which was used by both WhatsHap and SDhaP. WhatsHap was developed as a fixed-parameter tractable algorithm to solve the wMEC problem, where read coverage was the only fixed parameter [22]. SDhaP focused on finding an optimal solution to the MEC problem [21]. MAtCHap implemented the maximum allele co-occurrence objective function [24]. Finally, PEATH used the fitness function, which can identify good candidates for an optimization problem [16].

#### Input

Every HA algorithm had its own input format to reconstruct haplotypes, and these inputs varied as shown in Table 5. Three algorithms required matrices as input files, which differed from algorithm to algorithm. PEATH required two matrices as its input. The first was a quality score matrix, and the second was a sequence read matrix [16]. MAtCHap and SDhaP required one matrix. MAtCHap required one fragment matrix where the number of rows represented the reads, and the number of columns represented the heterozygous variants [24]. For SDhaP, the reads were arranged into a matrix according to their positions on the chromosome. These matrices were calculated by each HA algorithm. Users did not need to provide the matrices by themselves.

Another common input format was VCF and BAM. At least 2 algorithms such as HapCUT2 and WhatsHap required two input files, VCF and BAM, containing information on haplotype fragments and heterozygous variants [1, 22]. In addition to the input specifications mentioned above, there were also a few that were less commonly used among these papers. For example, MixSIH required aligned SNP fragment files for its input, and these fragments were retrieved through extraction from heterozygous alleles in aligned DNA fragments [14]. WhatsHap, along with VCF and BAM files, required sequencing reads from the individual sample [22]. Note, the input information was summarized based on what was stated in the 6 HA papers. In fact, when running these algorithms, the authors found that, except WhatsHap, the other 5 HA algorithms all required a similar DNA fragment file with some minor changes as shown in Fig. 2.

**Table 5:** Input files of 6 HA algorithms as stated in their papers or user manuals

Method	VCF	BAM	Matrix	Input
HapCUT2	X	X		Haplotype fragments (BAM files) and a list of heterozygous variants (VCF)
MixSIH				Aligned SNP fragments
PEATH			X	Two n by m matrices: M and Q (M = sequence read matrix; Q = quality score matrix)
WhatsHap	X	X		VCF file with variants of an individual and a BAM file with sequencing reads from that same individual
SDhaP			X	Reads were arranged into an m by n matrix according to positions on chromosome
MAtCHap			X	Uses one n by m fragment matrix where each row (n) represents reads and each column (m) represents the information of a heterozygous variant

The columns (from left to right) are HA methods, commonly used input formats (VCF file, BAM file, and Matrix), and input description. Each “X” represents the input format(s) that the given software package uses. The last column is a more detailed description of different input formats used in each algorithm

#### Comparison metrics

When comparing the 6 HA methods, the authors found that these methods used different metrics to compare with other algorithms. Then only commonly used ones were selected to compare different HA methods as listed in Table 6. Of all metrics, switch error rate and run time were used by all 6 HA methods, and MAtCHap used the greatest number of metrics. WhatsHap looked at three kinds of errors: flip errors, switch errors, and ambiguity errors. It also combined those three errors to find its total error. All HA methods used the switch error rate, and HapCUT2 and MAtCHap looked at the mismatch error rate. In addition, MAtCHap calculated its own total error rate by adding switch and mismatch errors.

**Table 6:** Comparison metrics

Method	Switch Error	Mismatch Error	MEC	Run time
HapCUT2	X	X		X
MixSIH	X			X
PEATH	X		X	X
WhatsHap	X			X
SDhaP	X		X	X
MAtCHap	X	X		X

The columns are (in order from left to right) haplotype assembly method, switch error, mismatch error, MEC, and run time. HA algorithms that use one of the four comparison metrics are marked with an “X”. Cells that are left blank mean that the specific algorithm in a row does not use this comparison metric

## Results

### Run time

**Fig. 3:**
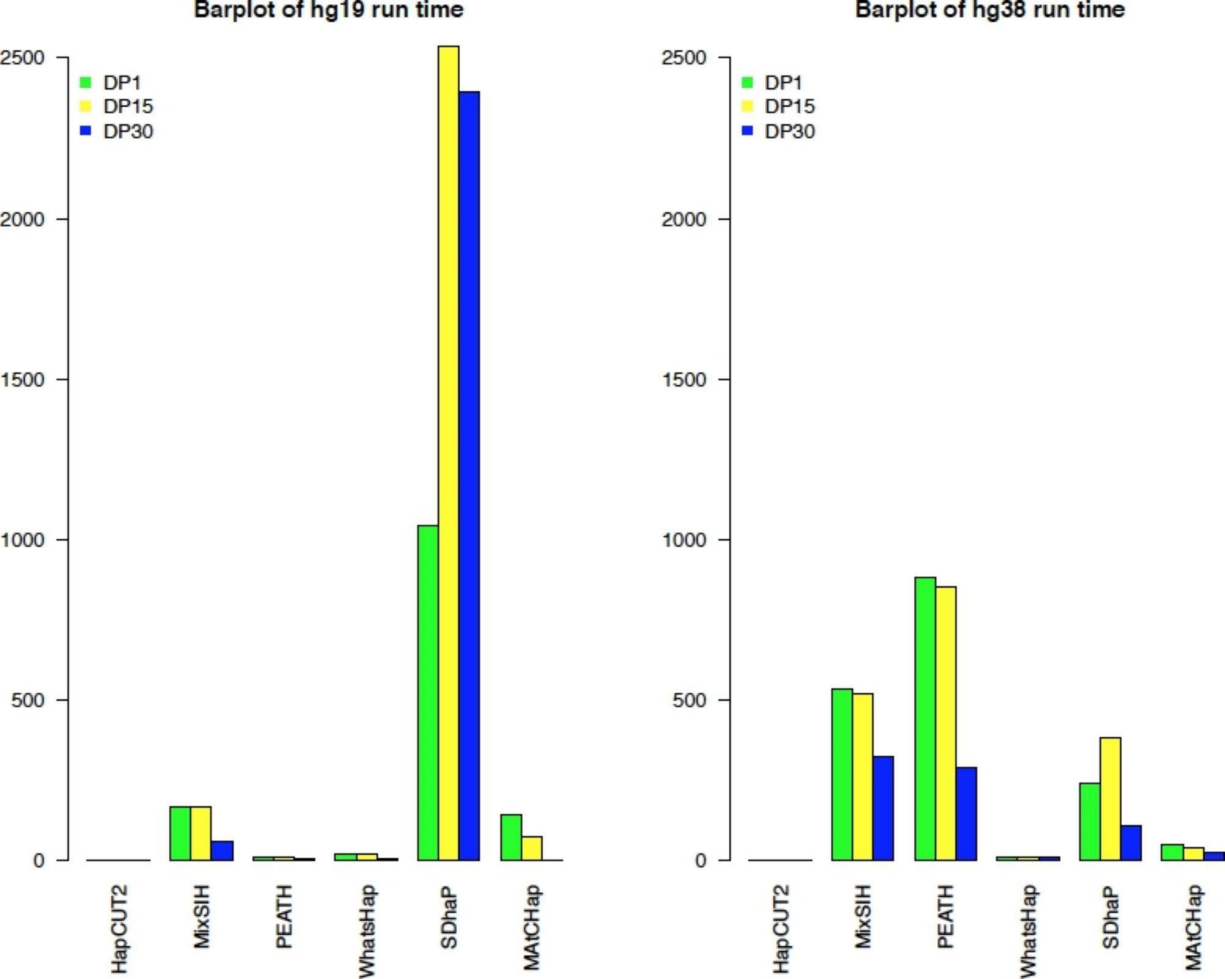
Run time of the 6 HA algorithms on hg19 and hg38 data. The vertical axis is in minutes

The run time of all 6 HA algorithms on 6 datasets (hg19 and hg38, each with 3 DP levels) was shown in Table 7; Fig. 3. Note, the run time was the CPU time used in executing each process, see detailed explanations in the Discussion. Table 7; Fig. 3 provided the same information, but they showed different details and perspectives. Table 7 gave specific run time, while Fig. 3 showed overall patterns. When comparing the run time of the 6 HA algorithms based on both hg19 and hg38 data’s 3 coverage levels, Table 7; Fig. 3 showed that the fastest HA was HapCUT2 for both hg19 and hg38 data. HapCUT2’s run time was consistently under 2 min. SDhaP took longer when running hg19 data than hg38 data (1042 ~ 2534 min for hg19 vs. 107 ~ 385 min for hg38). In fact, SDhaP was the slowest HA for hg19 but was near the average for hg38. Meanwhile, MixSIH and PEATH took longer when using hg38 data than hg19 data. Surprisingly, PEATH was the slowest HA for hg38, but it was one of the fastest ones (behind HapCUT2) for hg19. It took < 10 min for hg19 but 291 ~ 881 min for hg38. In general, Fig. 3 showed that the DP30 runs (blue bars) took significantly less time than DP1 (green bars) and DP15 (yellow bars). This difference might be because the input dataset of DP30 was much smaller after filtering based on coverage, i.e., with a much smaller number of DNA fragments as shown in Table 2. As the DP increased, the run time decreased (maybe because the dataset became smaller) or did not change much, but there were some outliers including SDhaP’s run time on both the hg19 and hg38 datasets.

When running the 6 HA algorithms across the hg19 and hg38 data, there seemed to be a longer run time for the hg38 dataset, especially for MixSIH and PEATH, but not for SDhaP. This longer time could be because hg38 sequencing reads were longer (2 × 150 bp paired-end) than the hg19 sequencing reads (2 × 100 bp paired-end). The run time increased from 1 to 2 s (HapCUT2) to 800 min (PEATH, DP1). Some outliers of the run time included WhatsHap (DP1 and DP15), MAtCHap (DP1 and DP15), and SDhaP, where the hg38 data run time was around 8 to 700 min faster than the hg19 data.

**Table 7:** Run time of the 6 HA algorithms on hg19 and hg38 data

no.HI hg19	DP1	DP15	DP30
HapCUT2	0m0.020s	1m27.862s	0m50.749s
MixSIH	166m53.561s	167m55.217s	60m32.406s
PEATH	9m9.623s	9m17.898s	6m55.111s
WhatsHap	21m47.595s	18m32.676s	7m47.223s
SDhaP	1042m19.795s	2534m53.175s	2395m2.418s
MAtCHap	145m32.562s	74m7.937s	2m5.477s
**no.HI hg38**	**DP1**	**DP15**	**DP30**
HapCUT2	1m38.330s	1m27.657s	1m15.984s
MixSIH	533m21.192s	521m52.663s	323m4.853s
PEATH	881m41.440s	851m6.780s	291m25.599s
WhatsHap	13m18.916s	13m23.951s	9m34.321s
SDhaP	243m21.180s	385m9.746s	107m9.087s
MAtCHap	47m46.312s	42m7.180s	23m57.059s

In each cell, “m” means minute; “s” means second. For example, “0m0.020s” means 0 min and 0.02 s. “no.HI” in the first column means that homozygous variants and indel sites were not included.

### Summary of block (blk) and SNV numbers

Table 8 showed the summary of block and SNV numbers for the hg19 and hg38 at the DP1 level. Each row had the summary of the number of SNV per block for all haplotype blocks inferred by one HA. This table showed that for the hg19 DP1 data, WhatsHap inferred about 50% more SNV positions (178,534 vs. 115,000). It had a much smaller number of blocks (10,132 vs. 32,000), but its blocks were generally longer than the other HA algorithms. WhatsHap’s longest block had 5,194 SNVs, while other HA algorithms’ longest blocks had only 770 SNVs. In the hg38 DP1 data, the total number of SNVs, blocks, and SNVs per block summary of WhatsHap was very similar to most other HA methods. In the hg38 DP1 data, PEATH and MAtCHap’s block lengths and numbers of SNVs were similar except for a small number of extremely long blocks. PEATH and MAtCHap’s longest blocks had 6,999 SNVs, but other HA methods’ longest blocks only had 2,100 ~ 2,400 SNVs. Similar outlying patterns of WhatsHap, PEATH, and MAtCHap could be found in hg19 and hg38’s DP15 and DP30 data, see Table 2 in Supplementary Material 1.

**Table 8:** Block and SNV number summary of the hg19 and hg38 DP1 data

hg19.DP1.no.HI	filterSNV (no”-“)	Block (no “-“)	Min	Q1	Median	Mean	Q3	Max
HapCUT2	115,215	32,150	2	2	2	3.58	4	770
MixSIH	115,790	32,252	2	2	2	3.59	4	770
PEATH	115,813	31,355	2	2	2	3.69	4	770
WhatsHap	178,523	10,132	2	2	2	17.6	9	5194
SDhaP	115,813	32,252	2	2	2	3.59	4	770
MAtCHap	115,813	31,355	2	2	2	3.69	4	770
**hg38 DP1.no.HI**	**filterSNV (no”-“)**	**Block (no “-“)**	**Min**	**Q1**	**Median**	**Mean**	**Q3**	**Max**
HapCUT2	100,686	21,710	2	2	2	4.64	5	2187
MixSIH	100,807	21,726	2	2	3	4.64	5	2190
PEATH	100,810	19,660	2	2	3	5.13	4	6988
WhatsHap	100,785	21,654	2	2	3	4.65	5	2479
SDhaP	100,810	21,726	2	2	3	4.64	5	2190
MAtCHap	100,810	19,660	2	2	3	5.13	4	6988

The top 7 rows are for the hg19 DP1 data. The bottom 7 rows are for the hg38 DP1 data. The first 3 columns are the HA names, total number of SNVs (after “-” being removed), and total number of haplotype blocks. “no.HI” in the first column means that homozygous variants and indel sites were not included. Columns 4 to 9 show a summary of the number of SNVs per block for all blocks listed in the third column. “Min” means minimum. “Max” means maximum. “Q1” is the 25th percentile. “Q3” is the 75th percentile

### Comparing with the “gold standard”

In order to compare the 6 HA algorithms, the authors first compared the chromosome positions from the known haplotypes with the SNV positions in the haplotypes inferred by each HA algorithm. That is, the comparison was first conducted with the so-called “gold standard,” i.e., a set of “high confidence” haplotypes of NA12878. The basic rationale was that if some (or many) SNV or chromosome positions inferred by these HA algorithms were not in the positions listed in the “gold standard” or known haplotypes, then it would not be meaningful to compare the haplotypes inferred by each HA with the so-called “gold standard” haplotypes. For example, HapCUT2 inferred 100 SNVs (or positions) in its output, and only 50 of them overlapped with the “gold standard” (or known haplotype) positions. It would not be meaningful to use the “gold standard” as a reference.

Table 9 showed that when the coverage increased (from 1X to 30X), some SNPs (or SNVs) with low coverage (e.g., < 10X) were filtered out from the haplotypes inferred by each HA method. Therefore, the SNP number decreased as the coverage filtering level increased. The decrease in the SNP (SNV) number in Table 9 could also be explained by Table 2, which showed that the number of DNA fragments (or sequencing reads) decreased as the filtering coverage level increased (from 1X to 15X and then to 30X).

**Table 9:** Comparing SNVs in 6 HA algorithms with positions in known haplotypes

HA methods	hg19.DP1	overlap	hg19.DP15	overlap	hg19.DP30	overlap
HapCUT2	115,215	43,868 (38.07%)	96,357	35,930 (37.29%)	18,824	3517 (18.68%)
MixSIH	115,790	44,210 (38.18%)	96,818	36,211 (37.40%)	18,885	3542 (18.76%)
PEATH	115,813	44,223 (38.18%)	96,835	36,225 (37.41%)	18,887	3542 (18.75%)
WhatsHap	178,523	97,865 (54.82%)	152,769	84,094 (55.05%)	24,628	7788 (31.62%)
WhatsHap.Filter	115,745	44,198 (38.19%)	96,783	36,204 (37.41%)	18,864	3540 (18.77%)
SDhaP.100,595	115,813	44,223 (38.18%)	96,835	36,225 (37.41%)	18,887	3542 (18.75%)
MAtCHap	115,813	44,223 (38.18%)	96,835	36,225 (37.41%)	18,887	3542 (18.75%)
**HA methods**	**hg38.DP1**	**overlap**	**hg38.DP15**	**overlap**	**hg38.DP30**	**overlap**
HapCUT2	100,686	84,197 (83.62%)	99,572	83,851 (84.21%)	63,970	52,185 (81.58%)
MixSIH	100,807	84,239 (83.56%)	99,675	83,887 (84.16%)	64,023	52,203 (81.54%)
PEATH	100,810	84,239 (83.56%)	99,676	83,888 (84.16%)	64,025	52,203 (81.54%)
WhatsHap	100,785	84,332 (83.68%)	99,648	83,983 (84.28%)	63,999	52,265 (81.67%)
WhatsHap.Filter	100,625	84,199 (83.68%)	99,487	83,846 (84.28%)	63,917	52,186 (81.65%)
SDhaP	100,810	84,239 (83.56%)	99,676	83,888 (84.16%)	64,025	52,203 (81.54%)
MAtCHap	100,810	84,239 (83.56%)	99,676	83,888 (84.16%)	64,025	52,203 (81.54%)

The columns are HA methods, numbers of SNVs obtained based on 3 DP level input data, and the corresponding overlap with 3,591,460 SNVs (for the hg19 data) and with 3,632,297 SNVs (for the hg38 data) in known haplotypes

By comparing based on the SNV positions only, as shown in Table 9, the authors found that for the hg19 data, except WhatsHap, only 37–38% (for DP1 and DP15) and 18% (for DP30) of SNVs in the other 5 HA algorithms overlapped with the known haplotype positions or SNVs. For the hg38 data, the overlap was much larger, 81–84%. There were still about 20% of SNV positions (roughly about 20,000 SNVs) that were not in the “gold standard” or known haplotypes. Therefore, it was not proper to compare their haplotypes with known ones.

### Pairwise comparison based on SNV and block

As shown in Table 8, the WhatsHap output for the hg19 DP1 data contained 178,523 SNVs, around 50% more than the other HA algorithms, which made it an “improper” or “unbalanced” comparison. In order to have a “proper” pairwise comparison, the authors filtered the WhatsHap output to only contain chromosome positions found in the PEATH and MAtCHap output files. These two algorithms were used because they had identical 115,813 SNV positions, and HapCUT2 and MixSIH had a similar number of SNVs. Pairwise comparisons were then conducted by including both the unfiltered and filtered WhatsHap (WhatsHap.Filter or WhatsHap.F) output with the other HA algorithms.

Table 10 was the pairwise comparison of 6 HA algorithms plus WhatsHap.Filter. Each row showed the pairwise comparison result with that row’s HA as the reference (HA1). For example, the second row in the top panel was HapCUT2. That is, the authors used its output as a reference, i.e., HA1. Then, the other HA algorithms listed in different columns were called HA2, where each HA2 was compared with HA1 to see how many blocks of HA2 (in the column) differ from HA1 (in the row). The comparison results were shown in the top panel (block disagreement), then the total number of SNVs was counted in these disagreement blocks. These comparison results were shown in the bottom panel (SNV disagreement). The authors conducted this type of pairwise comparison for hg19 and hg38’s DP1, DP15, and DP30 data. In total, there were 6 comparison tables, each with two panels, one for block disagreement and one for SNV disagreement as shown in Table 10. In order to avoid showing all these tables, the authors provided all pairwise comparison tables in Supplementary Material 2, which was an EXCEL file for hg19 and hg38 data and each with 3 sheets for DP1, DP15, and DP30 respectively.

Regarding the WhatsHap.Filter results, 115,745 out of 178,523 SNVs (after removing blocks with 1 SNV) were left. That is, WhatsHap and other HA algorithms had many common SNVs after the filter (selection). The filtered output was different from HapCUT2 in 2,362 out of 8,149 (i.e., 28.99%) blocks. These 2,362 blocks consisted of 94,685 SNVs. That is, on average, there were approximately 40 SNVs on each of these blocks. Thus, the output generated by WhatsHap usually had disagreements with other HA algorithms on long or large blocks. The authors also found that both the SNV and block disagreements were much lower for the WhatsHap.Filter output than those for the unfiltered output as shown in Table 10.

**Table 10:** Pairwise comparison based on block and SNV disagreements of hg19 DP1 data

Block-Disagree	HapCUT2	MixSIH	PEATH	WhatsHap	WhatsHap. Filter	SDhaP	MAtCHap
HapCUT2 (32,150 blocks)	0	459/32,150 (1.43%)	188/32,150 (0.58%)	1690/32,150 (5.26%)	1690/32,150 (5.26%)	4860/32,150 (15.12%)	494/32,150 (1.54%)
MixSIH (32,252 blocks)	885/32,252 (2.74%)	0	655/32,252 (2.03%)	1924/32,252 (5.97%)	1924/32,252 (5.97%)	5109/32,252 (15.84%)	664/32,252 (2.06%)
PEATH (31,355 blocks)	1005/31,355 (3.21%)	976/31,355 (3.11%)	0	2032/31,355 (6.48%)	2032/31,355 (6.48%)	5215/31,355 (16.63%)	838/31,355 (2.67%)
WhatsHap (10,132 blocks)	6353/10,132 (62.70%)	6344/10,132 (62.61%)	6328/10,132 (62.46%)	0		6602/10,132 (65.16%)	6347/10,132 (62.64%)
WhatsHap.Filter (8149 blocks)	2362/8149 (28.99%)	2258/8149 (27.71%)	2227/8149 (27.33%)	-	0	2824/8149 (34.65%)	2255/8149 (27.67%)
SDhaP (32,252 blocks)	5207/32,252 (16.14%)	5121/32,252 (15.88%)	5109/32,252 (15.84%)	5702/32,252 (17.68%)	5702/32,252 (17.68%)	0	5129/32,252 (15.90%)
MAtCHap (31,355 blocks)	1274/31,355 (4.06%)	954/31,355 (3.04%)	838/31,355 (2.67%)	2105/31,355 (6.71%)	2105/31,355 (6.71%)	5246/31,355 (16.73%)	0
**SNV- Disagree**	**HapCUT2**	**MixSIH**	**PEATH**	**WhatsHap**	**WhatsHap.** **Filter**	**SDhaP**	**MAtCHap**
HapCUT2 (115,215 SNVs)	0	8881/115,215 (7.71%)	6190/115,215 (5.36%)	15,960/115,215 (13.85%)	15,960/115,215 (13.65%)	27,098/115,215 (23.52%)	9224/115,215 (8.01%)
MixSIH (115,790 SNVs)	11,034/115,790 (9.53%)	0	10,153/115,790 (8.60%)	17,307/115,790 (14.95%)	17,307/115,790 (14.95%)	28,183/115,790 (24.34%)	10,240/115,790 (8.84%)
PEATH (115,813 SNVs)	12,872/115,813 (11.11%)	13,671/115,813 (11.80%)	0	19,263/115,813 (16.63%)	19,263/115,813 (16.63%)	31,494/115,813 (27.19%)	12,508/115,813 (10.80%)
WhatsHap (178,523 SNVs)	165,569/178,523 (92.74%)	165,603/178,523 (92.76%)	165,582/178,523 (92.75%)	0		166,693/178,523 (93.37%)	165,661/178,523 (92.80%)
WhatsHap.Filter (115,745 SNVs)	94,685/115,745 (81.80%)	93,576/115,745 (80.85%)	93,258/115,745 (80.57%)	-	0	96,750/115,745 (83.59%)	93,491/115,745 (80.77%)
SDhaP (115,813 SNVs)	28,693/115,813 (24.78%)	28,261/115,813 (24.40%)	28,253/115,813 (24.40%)	31,441/115,813 (27.15%)	31,441/115,813 (27.15%)	0	28,452/115,813 (24.57%)
MAtCHap (115,813 SNVs)	15,417/115,813 (13.31%)	13,563/115,813 (11.71%)	12,508/115,813 (10.80%)	19,750/115,813 (17.05%)	19,750/115,813 (17.05%)	31,801/115,813 (27.46%)	0

Each cell consists of the count and percentages of blocks (top panel) and SNVs (bottom panel) that two HA methods disagree with. “-” means that the authors do not compare WhatsHap with WhatsHap.Filter.

**Fig. 4:**
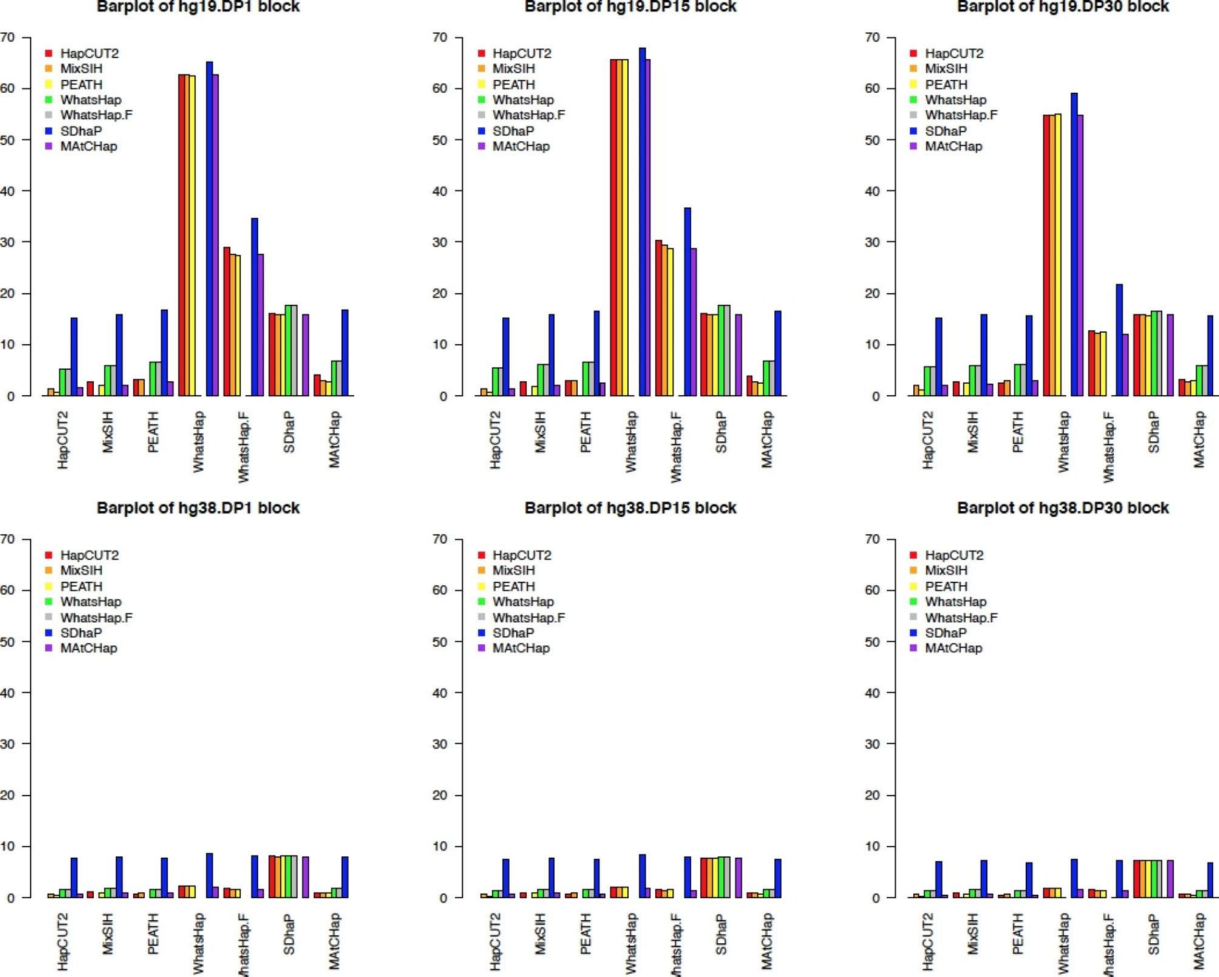
Bar plots of hg19 and hg38 block disagreement for pairwise comparisons of 6 algorithms. The vertical axis is the percentage of blocks that disagree between two HA algorithms (HA1 and HA2).

**Fig. 5:**
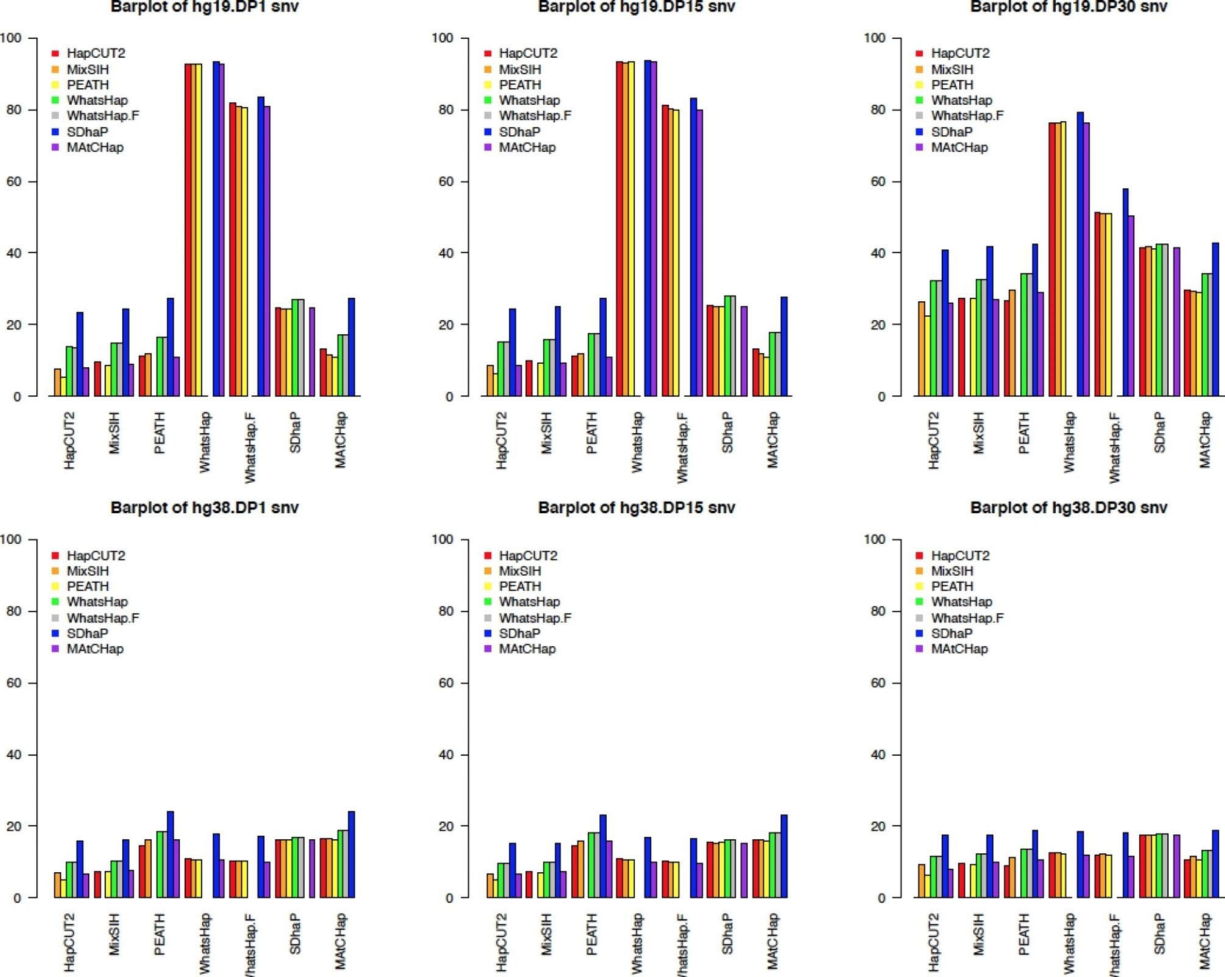
Bar plots of hg19 and hg38 SNV disagreement of 6 HA algorithms. The vertical axis is the percentage of SNVs that are in those disagreement blocks

To demonstrate the patterns in Table 10 clearly, bar plots were made, see Figs. 4 and 5. In these bar plots, in the bottom along the x-axis, 6 HA algorithms plus WhatsHap.Filter were shown as HA1, and the rainbow-colored bars were for each of the other HA algorithms, i.e., HA2, to compare. Figure 4 showed a few key patterns. First, the hg38 data (bottom 3 plots) showed that all 6 HA algorithms had much better agreement rates than the hg19 data (top 3 plots). Second, for both hg19 and hg38, comparisons with SDhaP as HA2 seemed to have the highest block disagreement rate, see the outstanding blue bars in Fig. 4. Third, for the hg19 data, WhatsHap, WhatsHap.Filter, and SDhaP had the highest block disagreement rate when used as HA1, see the x-axis for tall bar clusters in the top panel. However, for the hg38 data, only SDhaP generally had high disagreement rates, see the blue bars in the bottom 3 plots in Fig. 4. Pairwise comparisons were based on the total number of disagreement SNVs. Figure 5 showed similar patterns as Fig. 4. That is, for most comparisons, both hg19 and hg38 followed the same trend across three DP levels and had similar results, with hg19 having marginally higher SNV disagreement than hg38 (hg19 had around 10–20% while hg38 had less than 10% on average). With WhatsHap and WhatsHap.Filter as HA1, there was a much larger SNV disagreement rate than all other comparisons in the hg19 data, but not the hg38 data. Disagreement with SDhaP as HA2 was again the largest, see the blue bars in Figs. 4 and 5.

**Fig. 6:**
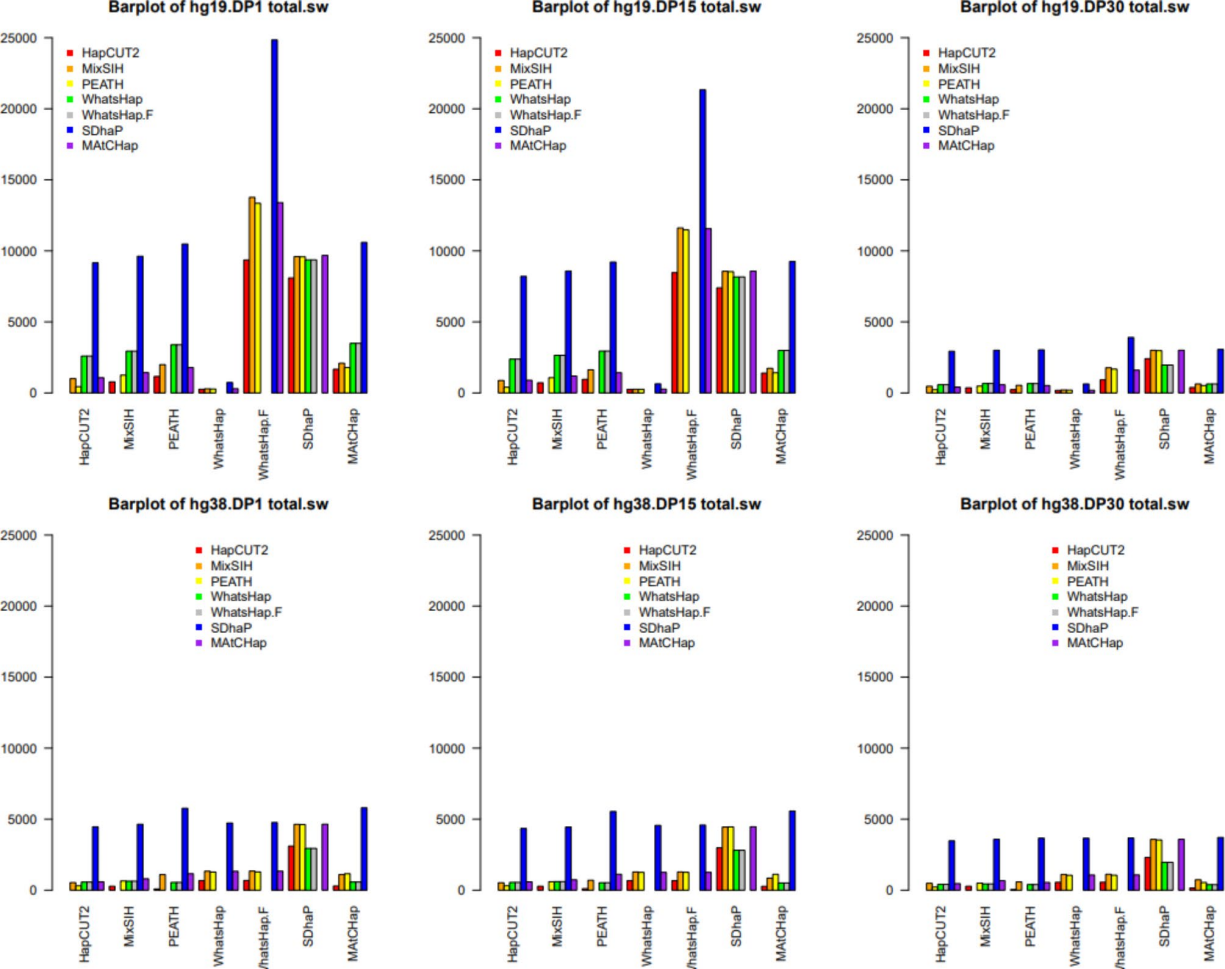
Bar plots of the total switches (total.sw) between each pair of algorithms

**Fig. 7:**
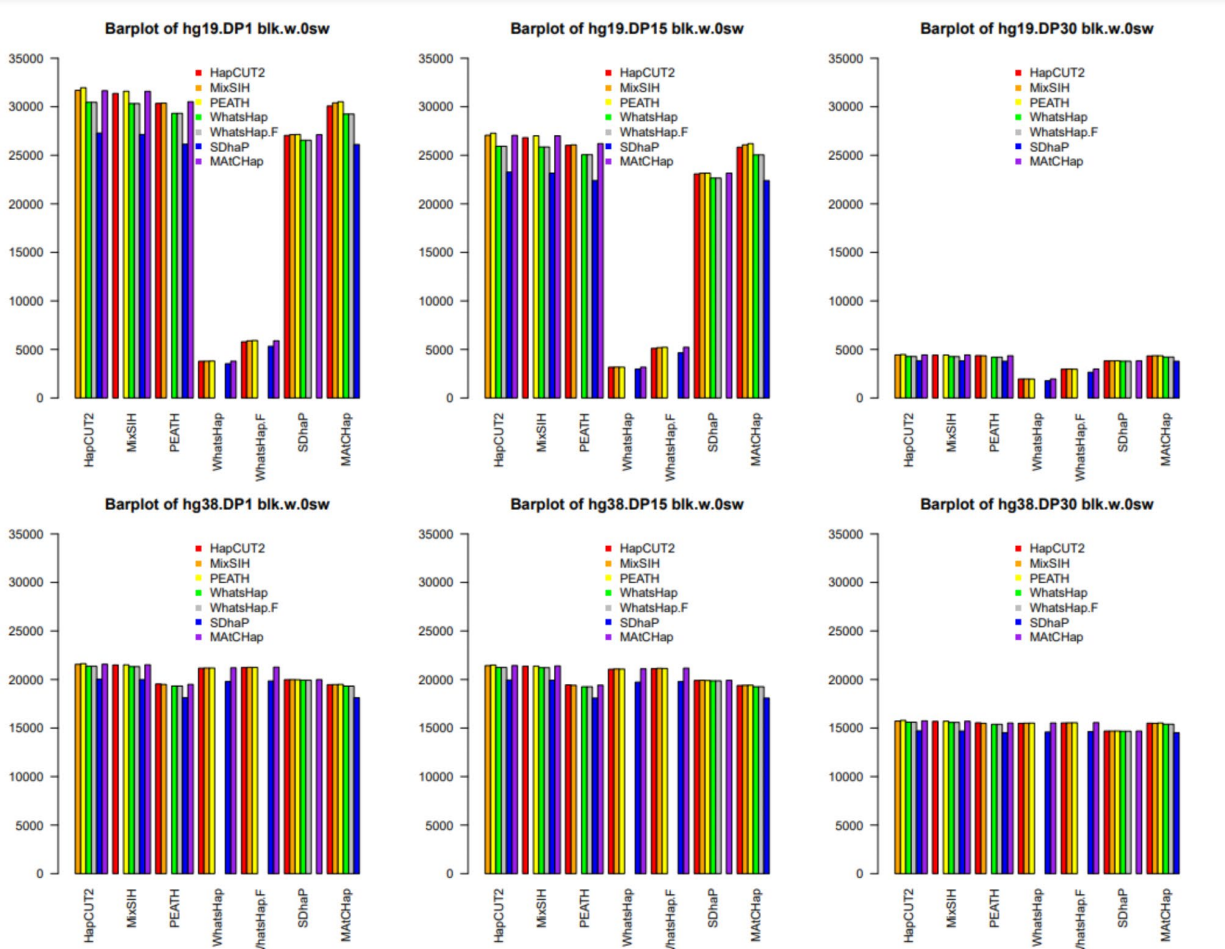
Bar plots of the number of blocks with zero switches (blk.w.0sw)

Similar to the pairwise comparison based on the block and SNV disagreement, the authors did the pairwise comparison using switch errors. As stated in the comparison analysis, 12 different but related switch-distance metrics were used to compare those HA algorithms. For each metric, the pairwise comparison results were plotted. Some of them had similar patterns and information. To avoid showing redundant figures, only 2 of these 12 metrics were shown. These two metrics were total.sw and blk.w.0sw as shown in Figs. 6 and 7 respectively. The bar plot layout (order and color) in these figures was the same as the one in Figs. 4 and 5. The bar plots of all 12 metrics were shown in Supplementary Material 3 (a PDF of 13 pages).

### Pairwise comparison based on switch distance

Figure 6 was for the metric total.sw. It showed the total number of switches a specific HA2 algorithm needed to match the HA1 haplotypes. This figure showed a few striking patterns. First, all 6 HA algorithms seemed to agree with each other better in the hg38 data than in the hg19 data. Second, for both hg19 and hg38 data, comparisons with SDhaP as HA2 showed the greatest difference (see blue bars that represent high disagreements), that is, more switches were needed. Third, for hg19, with WhatsHap.Filter and SDhaP as HA1, there were more switches (tall bar clusters). For hg38, only SDhaP had the largest number of switches (blue bars). Fourth, for the hg19 data, when WhatsHap was used as the HA1, the number of switches needed was the smallest (see the short bars above WhatsHap). This might be because WhatsHap had long blocks, while the other HA method (i.e., HA2) did not have those long blocks to compare with. Thus, it had a small number of switches needed. This could be seen in Fig. 7, which showed low bars above WhatsHap. However, for the WhatsHap.Filter, after removing those SNVs not inferred by other HA algorithms, its haplotype block structures were altered, and then many switches were needed. Fifth, for hg19 data, DP1 and DP15 plots were very similar in size. The switches required for DP30 were much smaller, see low bars in Fig. 6 for total switches.

Figure 7 was for the metric blk.w.0sw. This figure showed the total number of blocks with 0 switches. That is, the figure showed the number of blocks where those HA algorithms agreed with each other. This figure showed a few patterns. First, for the hg19 data, the blocks with 0 switches were consistently around 30,000 for DP1, 25,000 for DP15, and 5,000 for DP30, except when WhatsHap & WhatsHap.Filter was HA1 (see low bars). Second, for hg38, 6 HA algorithms’ block numbers were consistent with around 20,000 for DP1, 20,000 for DP15, and 15,000 for DP30. Third, the patterns for hg19 DP30 and hg38 DP1, DP15, and DP30’s numbers of blocks with 0 switches were similar. That is, 6 HA methods performed similarly when the DP level was high (i.e., DP30), and when the datasets had long reads (i.e., hg38 data).

WhatsHap had a much smaller number of blocks without switches (sw = 0) and with switches (sw > 0) (Figs. 6 and 7). This was because it had a significantly larger average number of SNVs per block that could not be checked due to different numbers of SNVs. In general, the number of SNV per block with switches was about 3 for hg19 and 4 for hg38. On average, for those blocks in which 2 HA methods disagreed with each other, 1 to 2 switches were necessary per short block.

Overall, HapCUT2, MixSIH, PEATH, and MAtCHap had relatively low disagreement percentages for both hg19 and hg38 datasets with different filtering levels. This conclusion can be made based on the pairwise comparison analyses of haplotype blocks, SNVs, and switch error rates. However, both SDhaP and WhatsHap resulted in much higher disagreement percentages with the other algorithms in the hg19 data. For hg38, WhatsHap had a similar performance when it was compared with HapCUT2, MixSIH, PEATH, and MAtCHap. SDhaP still performed differently.

## Discussion

### Coverage of DNA sequencing data

According to the original data sources (web pages) from which both the hg19 and hg38 datasets were downloaded, the sequencing coverage levels were at least 30X for both datasets. Examination of the chr10 data revealed that hg38 datasets had larger or better sequencing coverages. The hg19 datasets had a 25X median and 26.13X mean coverage, but hg38 datasets had a 33X median and 34.4X mean coverage. As for the hg19 data, 73.58% of the sites had more than 20X coverage; while for the hg38 data, 97.15% of the sites had more than 20X coverage. In addition to the sequencing-read-length difference, the coverage difference might be one of the reasons that those 6 HA algorithms performed differently.

### With and without homozygous variants and indels included

For all 6 HA algorithms, except MAtCHap, they could be run with or without homozygous variants and indels included. MAtCHap was developed to be run only on data with homozygous variants and indels removed. Therefore, the authors decided to run and report the results of all 6 HA algorithms with the homozygous variants and indels removed in this paper. Note that the authors did check the other 5 HA algorithms’ (except MAtCHap) outputs of these two versions: with and without homozygous variants and indels included. They found that these two versions were either identical or similar except some slight differences especially for MixSIH and SDhaP’s different runs, when examining the summary of the SNV and block numbers, as well as the pairwise comparison results. When there were some differences in the pairwise comparison, the comparison result based on no homozygous variants and indels was better and had lower disagreement rates.

### SDhaP

When running SDhaP, the authors found that it had an upper limit (700,000) for the number of lines in the input file as shown in its “SDhaP.c” script file. Thus, the NA12878 DNA fragment data must be split into two parts in order to use SDhaP. The authors found the location with the largest position difference that would still satisfy the limiting condition of SDhaP to split the input into two parts (e.g., 100,595 was used in the hg19 DP1 data). SDhaP’s running time was then reported as the total time of running these two parts. Note that users should also be aware that the SNV positions in SDhaP output were 0-based. These positions should be changed to be 1-based before comparing SDhaP with other HA algorithms. Furthermore, SDhaP tended to infer alleles as homozygous even though homozygous SNVs were removed from the input data. This might be one of the reasons that SDhaP disagreed with other HA algorithms. Other reasons contributing to SDhaP’s disagreement with other HA algorithms might be sequencing quality or coverage, uncertainty in the data, and limitations of the algorithm itself, as all HA algorithms had certain weaknesses.

### Ordering outputs

When checking the output files of 6 HA algorithms, the authors found that they were either sorted by SNV chromosome position or block number. Depending on how the file was sorted, this could affect the number of SNVs that were out of order. That is, the chromosome positions of SNVs in one specific block were not necessarily smaller than those in the next block. For example, block 1 had SNVs “1,2,5,” and block 2 had SNVs “3,7.” If the HA algorithm outputted files that were sorted by SNV position, the SNVs in these two blocks would read “1,2,3,5,7,” but the block IDs would be out of order, reading “1,1,2,1,2.” If the HA algorithm outputs were sorted by block ID and then SNV position, the block IDs would read “1,1,1,2,2,” but the SNVs would be out of order, reading “1,2,5,3,7.” The output for each of the 6 HA algorithms was ordered to have one file sorted by SNVs and another sorted by block ID. The authors found that the pairwise comparisons of their R and Perl scripts gave the same results, regardless of how the input file was sorted, but HA users should be very careful when examining the output of each HA algorithm. Additionally, the authors determined how each HA algorithm ordered its output by sorting and checking the output positions generated by all HA algorithms. The fact was that HapCUT2, MixSIH, and SDhaP’s output files were sorted by block ID (that is, their SNVs can be out of order), while WhatsHap’s was sorted by SNV position (that is, their block IDs could be out of order). Finally, PEATH and MAtCHap gave the cleanest outputs with both SNVs and block IDs well ordered.

### Limitations of this paper

There are a few limitations in this paper. First, haplotype assembly can be alignment-based or assembly-based [6]. In this paper, only alignment-based methods were compared. Second, some HA methods were developed for both diploid and polyploid haplotyping. The authors chose to use human data and only consider diploid haplotype assembly. Third, haplotype assembly can be done for both bulk sequencing and single cell sequencing data. Only the publicly available bulk sequencing datasets were used in this study. Fourth, although the authors have originally tried to run more than 20 different algorithms, only some of them could be run properly and compared for different practical reasons. Finally, many haplotype assembly methods or algorithms were developed [1, 10, 13, 14, 16, 21–24, 31, 33–43]. In particular, there were some methods developed for single-cell sequencing data [35, 39] or with a focus on long reads [33, 42–44]. However, there remain different challenges in haplotype assembly for repetitive regions, in scaling haplotype reconstruction efforts for routine applications, in validation, in benchmarking, and in annotation [7]. These have not been addressed in this paper. Instead, the authors narrowed down the research focus to simplify the study because haplotype assembly was indeed a complex research problem. Despite these limitations, this research work offered some useful and important inputs and perspectives to other users or bioinformaticians.

### NA12878 known haplotypes or the so-called “gold standard”

The NA12878 known (or true) haplotypes were obtained through various steps and processes as stated in Fig. 1 of Lowy-Gallego et al. 2019 [45]. These steps and processes were summarized below to help readers know their quality levels and understand how these haplotypes were obtained. Only Illumina sequence data with reads longer than 70 bp for WGS and 68 bp for whole exome sequencing (WES) data were used and aligned to GRCh38. The FASTQ files were converted to BAM files during the alignment process in which base quality scores were recalibrated and any duplicates were marked. The WGS and WES BAM files then underwent different methods of quality control. The BAM files were then used for variant identification using three established methods: GATK UnifiedGenotyper for WGS data, bcftools for both WGS and WES data, and Freebayes for WGS and WES data. This process produced 4 initial call sets that went under variant filtering. Each call set was normalized, and then various tools were used to decompose complex variants and re-sort and unify the remaining variants. Multiallelic sites were also discarded. Each of the 4 call sets was then processed into variant call format (VCF) files to generate consensus call sets. Eventually, the call set was filtered using a VariantScoreRecalibration method and phased using Beagle and SHAPEIT, producing the phased VCF files. VCF files with genomic likelihoods were divided into single files and then split into chunks that were processed in Beagle. Then, using SHAPEIT2, the genotypes and haplotypes were phased onto a highly accurate scaffold. The scaffold was also created by SHAPEIT2 using available array data from Illumina Omni 2.5 or Affymetrix 0.6 data from the 1000 Genomes Project.

### About comparing with a “gold standard”

To provide a valid comparison, the authors have used a “gold standard,” i.e., a set of “high confidence” known (or inferred) haplotypes of NA12878. “High confidence” often means “high coverage” and/or “high quality.” That is, a lot of uncertain positions (SNVs) with low sequencing coverages or qualities were not included in the final “gold standard.” In fact, there was more than one version of the “gold standard,” although the authors only used one version in this paper. The one used in this paper was better than the one that was not mentioned. They all had only about 30 ~ 40% of positions overlapped with the SNV positions inferred for the hg19 DP1 data. The authors chose to use the current one because it was the best NA12878 haplotype data that could be found so far. This “gold standard” haplotype dataset had about 80% of overlap with the SNV positions inferred by each HA method in the hg38 DP1 data. The low overlap percentages (30 ~ 40% for hg19 DP1 data and 80% for hg38 DP1 data) were due to various reasons. First, no matter how accurate a sequencing method was, a sequencing dataset could still have some errors, and some genomic regions (or positions) could be sequenced with uncertainty or low quality. Second, SNV calling and haplotype assembly have been very complex problems that involve multiple steps and processes as stated in Lowy-Gallego et al. 2019 [45]. The whole process underwent many different methods and steps of quality control to ensure a “high confidence.” As shown in Table 9, even filtering only based on one factor (DNA sequencing coverage), the number of remaining positions decreased significantly. Thus, only a certain percentage of positions (definitely not 100%) were included in the final “high confidence” true haplotypes (or “gold standard”). Therefore, if the haplotypes inferred by each HA were compared with the “high confidence gold standard” haplotypes, many SNVs would have to be removed from the inferred haplotypes. Then, the “block structure” from each HA would be destroyed. This kind of comparison was not meaningful for this study. Therefore, the authors focused on pairwise comparisons in this paper to provide HA users with some new perspectives and results.

#### Strengths and weaknesses of 6 HA algorithms

All 6 HA methods had certain strengths, weaknesses, and unique features. Below were a few typical ones. First, HapCUT2, MixSIH, PEATH, and MAtCHap were all probability-based methods. This might be the main reason they performed similarly. Second, HapCUT2 could work across various sequencing technologies: fosmid-based dilution pool, 10X genomics linked-read, PacBio SMRT, and Hi-C. Other HA algorithms might not be able to produce results for data on all aforementioned sequencing types [1]. Third, HapCUT2 included extractHAIRs, a convenient tool available for other software packages. WhatsHap was easy to use as it only required one VCF file and one BAM file. It did not require a fragment file to run. However, the version of WhatsHap that was used in this study required Python 3.6 or above, which could be installed directly by running pip (a Python command) or conda (a Miniconda command). Some inexperienced users might find it difficult to get these packages installed properly in a Linux server. Fourth, as for the run time, HapCUT2 and WhatsHap had stable performance across 6 datasets; but MixSIH, PEATH, and SDhaP had more variation. HapCUT2 and WhatsHap were not affected by read length. They can deal with the increasing read lengths with future sequencing technologies. Fifth, the strength of SDhaP was that it can do both diploid and polyploid haplotype assembly [21] although this study did not compare polyploid HA algorithms. Polyploid haplotyping is more challenging than diploid haplotyping as the complexity of the problem increases drastically for polyploid genomes [7]. Sixth, WhatsHap and SDhaP were MEC-based methods. Motazedi et al.’s simulation study showed that this type of method had an inherent problem because MEC was sensitive to local similarities between homologues [46]. This sensitivity led to approximately identical MEC scores for several different HA algorithms, causing a suboptimal solution to be reported. Other authors mentioned similar problems of MEC-based methods too [33, 44]. Seventh, both the authors of this paper and the PEATH authors found that some SNV positions in the SDhaP outputs were homozygous while these positions were indeed heterozygous in the gold standard dataset [16]. In addition, PEATH authors also mentioned that although SDhaP had certain advantages, its results had a large variance [16].

### About run time and settings

All HA algorithms were run with a similar command line “time HA.algorithm input output”. The input and output settings were summarized in Table 11. This table showed that an additional parameter was used for MixSIH, i.e., “-a 0.05”, which was required by this algorithm, and this setting was close to the default value (0.1). No other parameters were set up for the other 5 algorithms. The Unix command “time” was used to obtain the CPU time of each job. This command provided three different time measurements: Real, User, and System. The Real time is wall clock time, that is, the time from the beginning to the end of the call including time used by other processes and time the process spends blocked. The User time is the actual CPU time used in executing the process. The System time is the amount of CPU time spent in the kernel within the process. All these measurements were recorded when running 6 algorithms on both hg19 and hg38 data as shown in the Supplementary Material 4 (an Excel file with two sheets, one for hg19 and one for hg38 data). In general, the “User + System” time should be the total CPU time. In this paper, only the User time was reported as the CPU time in Table 7. The System time was not included as part of the CPU time in Table 7 because the amount of the System time was so small (around 1 min or less) for all HA algorithms except SDhaP’s hg19 DP15 and DP30 runs. A record of low System time suggested the application was running efficiently, but a record of high System time could be an indicator of executing inefficiencies and possible issues of a specific algorithm. Because SDhaP had an outlying performance as documented in the previous subsection “SDhaP”, its high System time (hg19 data) could be due to some possible issues only related to itself. In addition, whether including the System time or not would not affect the key conclusion of comparing the running time. Therefore, for the sake of consistency and simplicity, only the actual CPU time used in executing each process (i.e., User time) was reported in Table 7.

**Table 11:** The specific input, output, and other key parameters used to run each algorithm

Algorithm	Input	Output	Other parameters
HapCUT2	--fragments DNA.fragment.file --vcf variant.VCF.file	--output output.file.txt	None
MixSIH	Sorted DNA fragment file	output.file name	- a 0.05
PEATH	DNA fragment file	output.file name	None
WhatsHap	Sorted BAM file	output.file name	None
SDhaP	DNA fragment file	output.file name	None
MAtCHap	-F sorted.fragment.file V variant.VCF.file	-L output.label -O output.folder	None

All HA jobs were run sequentially without using parallel computing settings. In addition, the run time was the time that each HA algorithm used to reconstruct haplotypes. The input preparation time (e.g., getting a DNA fragment file or a VCF file as shown in Fig. 2) for each HA algorithm was not included in the run time reported in this paper. The input preparation time could range from minutes to hours depending on the data size. Finally, the authors of certain HA methods (e.g., WhatsHap) kept improving their algorithms by adding new features over the last several years. It is possible that other researchers used less (or more) time if they installed a different version of a specific algorithm. For the sake of comparison, readers may check the algorithm version or software installation time listed in the last column of Table 1.

#### About SNVs and haplotype blocks

The terms “SNVs” and “haplotype blocks” have been commonly used in many recent haplotype assembly papers. Before the existence of the next (or second) generation sequencing (NGS) and third generation (or single cell) sequencing (TGS), the term “haplotype” was mainly defined as the alleles of consecutive SNPs in the nearby region on the same chromosome. The distance between the first and last SNPs on a haplotype block could be as large as several thousand bases to 100 thousand bases as shown in Fig. 2 of Daly et al. 2001 [47]. The term haplotype block was inherited or commonly used in the new field of haplotype assembly. However, the length of a haplotype block shown in the output of a haplotype assembly algorithm could be just 100 bases or less. These haplotype blocks consisted of some SNVs that were not validated as SNPs yet. That is, with the development of DNA sequencing technologies, the meaning of haplotype blocks used in the haplotype assembly output somehow has evolved or become slightly different from the original one defined before the existence of NGS and TGS. These terms were explained here for the sake of clarification.

#### The scientific value and the goal of this study

Comparative analysis of existing algorithms may not be considered as innovative as a new HA algorithm, but it can address recurring questions. The current situation is that although many HA algorithms have been developed, bioinformaticians may not know which one to use and how well these algorithms perform. The authors were often asked these questions when attending conferences. Therefore, in this paper, a comparison study was conducted to answer some important questions that involved challenging bioinformatic data analysis. This study answered some questions that were not well addressed before, and the findings shown in this paper can help many users. This research work showed how different HA methods might perform if other researchers analyzed a newly generated dataset (or a publicly available dataset) that had no known haplotypes (i.e., the so-called “gold standard”). This study has also offered different perspectives for users to investigate with their own data and explain what they might find (e.g., filtering based on different coverage levels). The findings of this study have not been reported by other authors yet. Therefore, this research work is novel and useful. Two practical examples of previous publications on comparing a few SNP calling algorithms [48] and alignment algorithms [49] have been cited many times by others. These citations can show the scientific value of comparison analysis. Therefore, this study is original and valuable.

## Conclusion

In this paper, the pairwise comparative analysis of 6 HA methods was conducted. Two publicly available sequencing datasets, hg19 and hg38 from one sample (NA12878), were used for the comparative analysis. The aligned reads were downloaded and then filtered using 3 sequencing depth (DP) levels, DP1, DP15, and DP30 corresponding to ≥ 1X, ≥ 15X, and ≥ 30X coverage. This filtering step was done for each of the two datasets (hg19 and hg38). The 6 HA algorithms were run on these 6 datasets and their performances were then compared. The fastest HA was HapCUT2 for both hg19 and hg38. Its run time was consistently under 2 min. In addition, WhatsHap was relatively fast, and its run time was 21 min or less for all 6 datasets. The other 4 HA algorithms’ run time varied across different datasets and DP filtering levels. A publicly available known haplotype dataset was used to compare the SNV positions of these known haplotypes with the SNV inferred in each of the 6 HA output files. The finding was that < 40% of SNVs inferred in the hg19 output overlapped with known haplotypes, and only 81–84% of hg38 output overlapped with the SNV positions in the known haplotypes. Then pairwise comparisons were conducted to see how well the 6 HA methods agreed with each other by comparing their haplotype blocks, SNVs, and switch distances. The comparison results showed that for hg19 data, HapCUT2, MixSIH, PEATH, and MAtCHap had relatively large agreement levels, but WhatsHap and SDhaP had outlying performances. For the hg38 data, all algorithms, except SDhaP, performed similarly. The pairwise comparison results based on the sequencing datasets of the same sample could help users to better understand current HA methods. This study also provided useful insights on developing more accurate and efficient methods.

## Electronic supplementary material

Below is the link to the electronic supplementary material.

Supplementary Material 1

Supplementary Material 2

Supplementary Material 3

Supplementary Material 4

## Data Availability

The original or raw datasets supporting the conclusions of this article were publicly available with the ftp sites listed below. The datasets and analysis results supporting the conclusions of this article were included within the article and its supplementary materials. The two DNA sequencing datasets (hg19 and hg38) were downloaded from these web pages: ENA-hg19: ftp://ftp.sra.ebi.ac.uk/vol1/run/ERR262/ERR262997/NA12878_S1.bam. ENA-hg38: ftp://ftp.sra.ebi.ac.uk/vol1/run/ERR323/ERR3239334/NA12878.final.cram. The known (or true) haplotype dataset of NA12878 was downloaded using the following ftp address: ftp://ftp.1000genomes.ebi.ac.uk/vol1/ftp/data_collections/1000_genomes_project/release/20190312_biallelic_SNV_and_INDEL/ALL.chr10.shapeit2_integrated_snvindels_v2a_27022019.GRCh38.phased.vcf.gz.

## List of abbreviations

BAM: Binary Alignment Map
BCF: BIM Collaboration Format
DP: Depth (Sequencing depth)
ENA: European Nucleotide Archive
HA: Haplotype Assembly
NGS: Next Generation Sequencing
TGS: Third Generation Sequencing
SNV: Single Nucleotide Variant
SNP: Single Nucleotide Polymorphism
VCF: Variant Call Format
WGS: Whole Genome Sequencing
WES: Whole Exome Sequencing
