# Supplementary material for: Pairwise comparative analysis of six haplotype assembly methods based on users’ experience
Source: BMC Genom Data. 2023 Jun 29;24:35. doi: 10.1186/s12863-023-01134-5 (PMC10311811; doi:10.1186/s12863-023-01134-5)
Supplement: Supplementary file 1 — Supplementary Material 1 [file 12863_2023_1134_MOESM1_ESM.docx]

**Additional File 1: Example output files and summary of haplotype blocks and SNVs**

Additional File 1 includes Supplemental Tables 1 and 2 for the following manuscript

**Pairwise comparative analysis of six haplotype assembly methods based on users’ experience**

Shuying Sun, Flora Cheng, Daphne Han, Sarah Wei, Alice Zhong, Sherwin Massoudian, Alison B. Johnson

**Supplemental Table 1: Raw output examples of 6 haplotype assembly algorithms.**

| ==> HapCUT2raw output <==  3 0 1 chr10 69071 G A 0/1:88,0,55 0 . 100.00 1 BLOCK: offset: 3 len: 2 phased: 2 SPAN: 12 fragments 6  4 0 1 chr10 69083 C T 0/1:106,0,106 0 . 100.00 1 BLOCK: offset: 3 len: 2 phased: 2 SPAN: 12 fragments 6  10 0 1 chr10 76210 A G 0/1:136,0,67 0 . 100.00 2 BLOCK: offset: 10 len: 2 phased: 2 SPAN: 84 fragments 3 |
| --- |
| ==> PEATH raw output <==  3 1 0 1 Block Number: 1 Block Length: 2 Phased Length: 2 Number  of Reads: 6 Start position: 3 Weighted MEC: 0.0433981 MEC: 0  4 1 0 1 Block Number: 1 Block Length: 2 Phased Length: 2 Number  of Reads: 6 Start position: 3 Weighted MEC: 0.0433981 MEC: 0  10 1 0 2 Block Number: 2 Block Length: 2 Phased Length: 2 Number  of Reads: 3 Start position: 10 Weighted MEC: 0.00247231 MEC: 0 |
| ==> MixSIH raw output <==  3 0 1 1 BLOCK: offset: 3 len: 2 phased: 2  4 0 1 1 BLOCK: offset: 3 len: 2 phased: 2  10 0 1 2 BLOCK: offset: 10 len: 2 phased: 2 |
| ==> SDhaP raw output <==  Block 1 Length of haplotype block 2 Number of reads 6 Total MEC 0  2 1 0  3 1 0  Block 2 Length of haplotype block 2 Number of reads 3 Total MEC 0  7 1 0  8 1 0 |
| ==> MAtCHap raw output (VCF format) <==  #CHROM POS ID REF ALT QUAL FILTER INFO FORMAT NA12878  chr10 66957 . C T 32.00970 . DP=25;VDB=0.169891;SGB=-0.651104;RPB=0.0979828;MQB=0.68264;MQSB=0.0856131;BQB=0.998305;MQ0F=  0.2;AF1=0.5;AC1=1;DP4=6,11,5,3;MQ=21;FQ=35.0165;PV4=0.389169,1,1,0.0642211 GT:MS:BID 0/1:NA:0  chr10 67774 . G C 30.01130 . DP=10;VDB=0.789602;SGB=-0.556411;RPB=0.727822;MQB=0.335918;MQSB=1;BQB=0.895781;MQ0F=0.3;AF1=  0.50001;AC1=1;DP4=6,0,3,1;MQ=26;FQ=16.9948;PV4=0.4,0.456698,1,0.23351 GT:MS:BID 0/1:NA:0  chr10 69071 . G A 58.00730 . |
| ==> WhatsHap raw output (VCF format) <==  #CHROM POS ID REF ALT QUAL FILTER INFO FORMAT NA12878  chr10 67774 . G C 30.0113 . DP=10;VDB=0.789602;RPB=0.727822;MQB=0.335918;BQB=0.895781;MQSB=1.0;SGB=-0.556411;MQ0F=0.3;AF1=0.50001;AC1=1.  0;MQ=26;FQ=16.9948;PV4=0.4,0.456698,1.0,0.23351;DP4=6,0,3,1 GT:PL 0/1:60,0,44  chr10 69071 . G A 58.0073 . DP=15;VDB=0.0476385;RPB=0.0289435;MQB=0.269146;BQB=0.87319;MQSB=0.941512;SGB=-0.636426;MQ0F=0.2;AF1=0.500001  ;AC1=1.0;MQ=21;FQ=28.0197;PV4=0.61927,1.0,1.0,1.0;DP4=3,5,4,3 GT:PL:PS 1\|0:88,0,55:66957 |

Supplemental Table 1 shows that HapCUT2, PEATH, MixSIH, and SDhaP have similar output formats. The first column is the SNV index based on the input VCF file. Note that SDhaP’s first SNV index is 0-based, but HapCUT2, PEATH, MixSIH are 1-based. The second and third columns are the haplotypes on two chromosomes. The other columns are detailed information. Note that the output of each SNV position or line is a bit long for HapCUT2and PEATH. Therefore, they are put in the next row. The MAtCHap and WhatsHap raw output files are in VCF files. In these output examples, the header lines of their VCF output files are removed.

**Supplemental Table 2: SNV and block summary of hg19 and hg38 data of 3 depth (DP) levels.**

| **hg19 DP1.no.HI** | **filterSNV (no"-")** | **Blk (no "-")** | **Min** | **Q1** | **Median** | **Mean** | **Q3** | **Max** |
| --- | --- | --- | --- | --- | --- | --- | --- | --- |
| HapCUT2(No length 1 blk) | 115215 | 32150 | 2 | 2 | 2 | 3.584 | 4 | 770 |
| MixSIH | 115790 | 32252 | 2 | 2 | 2 | 3.59 | 4 | 770 |
| PEATH | 115813 | 31355 | 2 | 2 | 2 | 3.694 | 4 | 770 |
| WhatsHap (No length 1 blk) | **178523** | **10132** | 2 | 2 | 2 | 17.62 | 9 | **5194** |
| SDhaP - 100595 split | 115813 | 32252 | 2 | 2 | 2 | 3.591 | 4 | 770 |
| MAtCHap | 115813 | 31355 | 2 | 2 | 2 | 3.694 | 4 | 770 |
| **hg19 DP15.no.HI** | **filterSNV (no"-")** | **Blk (no "-")** | **Min** | **Q1** | **Median** | **Mean** | **Q3** | **Max** |
| HapCUT2(No length 1 blk) | 96357 | 27449 | 2 | 2 | 2 | 3.51 | 4 | 775 |
| MixSIH | 96818 | 27537 | 2 | 2 | 2 | 3.516 | 4 | 775 |
| PEATH | 96835 | 26837 | 2 | 2 | 2 | 3.608 | 4 | 775 |
| WhatsHap (No length 1 blk) | **152769** | **9237** | 2 | 2 | 4 | 16.54 | 10 | **3117** |
| SDhaP - 100595 split | 96835 | 27537 | 2 | 2 | 2 | 3.517 | 4 | 775 |
| MAtCHap | 96835 | 26,837 | 2 | 2 | 2 | 3.608 | 4 | 775 |
| **hg19 DP30.no.HI** | **filterSNV (no"-")** | **Blk (no "-")** | **Min** | **Q1** | **Median** | **Mean** | **Q3** | **Max** |
| HapCUT2(No length 1 blk) | 18824 | 4529 | 2 | 2 | 2 | 4.156 | 4 | 773 |
| MixSIH | 18885 | 4540 | 2 | 2 | 2 | 4.16 | 4 | 773 |
| PEATH | 18887 | 4479 | 2 | 2 | 2 | 4.217 | 4 | 773 |
| WhatsHap (No length 1 blk) | **24628** | **4338** | 2 | 2 | 3 | 5.677 | 5 | **3428** |
| SDhaP - 100595 split | 18887 | 4540 | 2 | 2 | 2 | 4.16 | 4 | 773 |
| MAtCHap | 18887 | 4479 | 2 | 2 | 2 | 4.217 | 4 | 773 |

| **hg38 DP1.no.HI** | **filterSNV (no"-")** | **Blk (no "-")** | **Min** | **Q1** | **Median** | **Mean** | **Q3** | **Max** |
| --- | --- | --- | --- | --- | --- | --- | --- | --- |
| HapCUT2(No length 1 blk) | 100686 | 21710 | 2 | 2 | 3 | 4.638 | 5 | 2187 |
| MixSIH | 100807 | 21726 | 2 | 2 | 3 | 4.64 | 5 | 2190 |
| PEATH | 100810 | 19660 | 2 | 2 | 3 | 5.128 | 4 | **6988** |
| WhatsHap (No length 1 blk) | 100785 | 21654 | 2 | 2 | 3 | 4.654 | 5 | 2479 |
| SDhaP - 100595 split | 100810 | 21726 | 2 | 2 | 3 | 4.64 | 5 | 2190 |
| MAtCHap | 100810 | 19660 | 2 | 2 | 3 | 5.128 | 4 | **6988** |
| **hg38 DP15.no.HI** | **filterSNV (no"-")** | **Blk (no "-")** | **Min** | **Q1** | **Median** | **Mean** | **Q3** | **Max** |
| HapCUT2(No length 1 blk) | 99572 | 21564 | 2 | 2 | 3 | 4.617 | 4 | 2187 |
| MixSIH | 99675 | 21578 | 2 | 2 | 3 | 4.619 | 4 | 2190 |
| PEATH | 99676 | 19558 | 2 | 2 | 3 | 5.096 | 4 | **6953** |
| WhatsHap (No length 1 blk) | 99648 | 21511 | 2 | 2 | 3 | 4.632 | 4 | 2483 |
| SDhaP - 100595 split | 99676 | 21578 | 2 | 2 | 3 | 4.619 | 4 | 2190 |
| MAtCHap | 99676 | 19558 | 2 | 2 | 3 | 5.096 | 4 | **6953** |
| **hg38 DP30.no.HI** | **filterSNV (no"-")** | **Blk (no "-")** | **Min** | **Q1** | **Median** | **Mean** | **Q3** | **Max** |
| HapCUT2(No length 1 blk) | 63970 | 15813 | 2 | 2 | 3 | 4.045 | 4 | 2001 |
| MixSIH | 64023 | 15821 | 2 | 2 | 3 | 4.047 | 4 | 2005 |
| PEATH | 64025 | 15594 | 2 | 2 | 3 | 4.106 | 4 | **4415** |
| WhatsHap (No length 1 blk) | 63999 | 15778 | 2 | 2 | 3 | 4.056 | 4 | 2434 |
| SDhaP - 100595 split | 64025 | 15821 | 2 | 2 | 3 | 4.047 | 4 | 2005 |
| MAtCHap | 64025 | 15594 | 2 | 2 | 3 | 4.106 | 4 | **4415** |

Supplemental Table 2 is a more comprehensive summary corresponding to Table 8 in the main manuscript. The outlying patterns of WhatsHap, PEATH, and MAtCHap are highlighted in bold and underlined. That is, for hg19 DP1, DP15, and DP30 datasets, WhatsHap infers about 30% to 50% more SNV positions and it has much smaller number of blocks, but its block length is generally longer than the other HA algorithms. In the hg38 DP1, DP15, and DP30 data, PEATH and MAtCHap infer similar numbers of SNVs and block lengths are similar except a small number of extremely long blocks.
