# Supplementary material for: Pairwise comparative analysis of six haplotype assembly methods based on users’ experience
Source: BMC Genom Data. 2023 Jun 29;24:35. doi: 10.1186/s12863-023-01134-5 (PMC10311811; doi:10.1186/s12863-023-01134-5)
Supplement: Supplementary file 3 — Supplementary Material 3 [file 12863_2023_1134_MOESM3_ESM.pdf]

Additional File 3

Supplemental Figure 1: Switch distance pairwise comparison bar plots.

This figure consists of 12 sub-figures on 12 pages. These 12 sub-figures are for the following 12 different switch distance metrics: blk.w.0sw, blk.w.NAsw, blk.w.sw, snv.in.blk.w.0sw, snv.in.blk.w.NAsw, snv.in.blk.w.sw, snv.per.blk.w.0sw, snv.per.blk.w.NAsw, snv.per.blk.w.sw, total.sw, snv.by.sw, and sw.per.blk. In each sub-figure, the top panel is for the hg19 data with 3 sequencing coverage or depth (DP) levels (DP1, DP15, DP30), and the bottom panel is for the hg38 data with 3 depth levels (DP1, DP15, DP30).

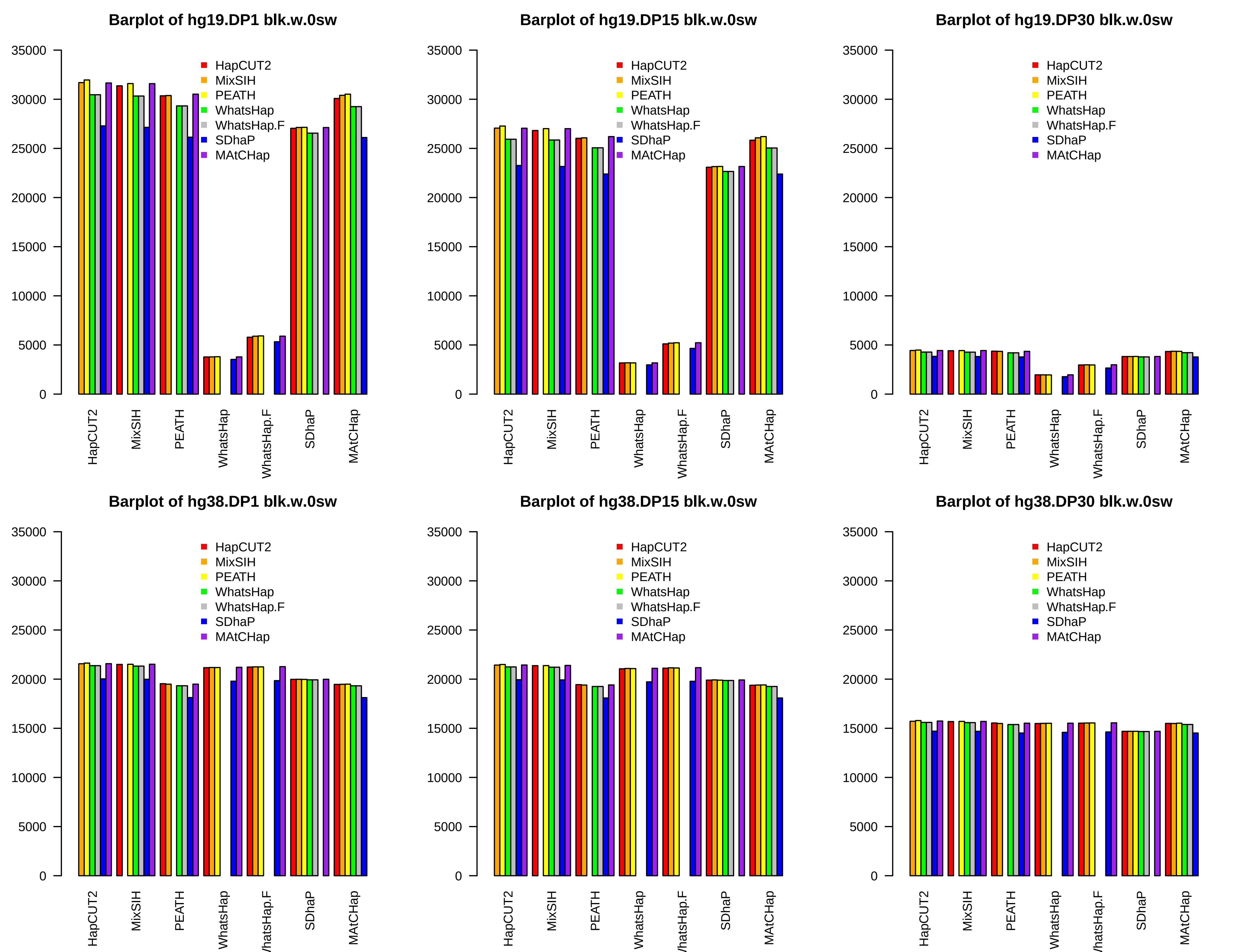
Barplot of hg19.DP1 blk.w.0sw
Barplot of hg19.DP15 blk.w.0sw
Barplot of hg19.DP30 blk.w.0sw
Barplot of hg38.DP1 blk.w.0sw
Barplot of hg38.DP15 blk.w.0sw
Barplot of hg38.DP30 blk.w.0sw
0
5000
10000
15000
20000
25000
30000
35000
HapCUT2
MixSIH
PEATH
WhatsHap
WhatsHap.F
SDhaP
MAtCHap

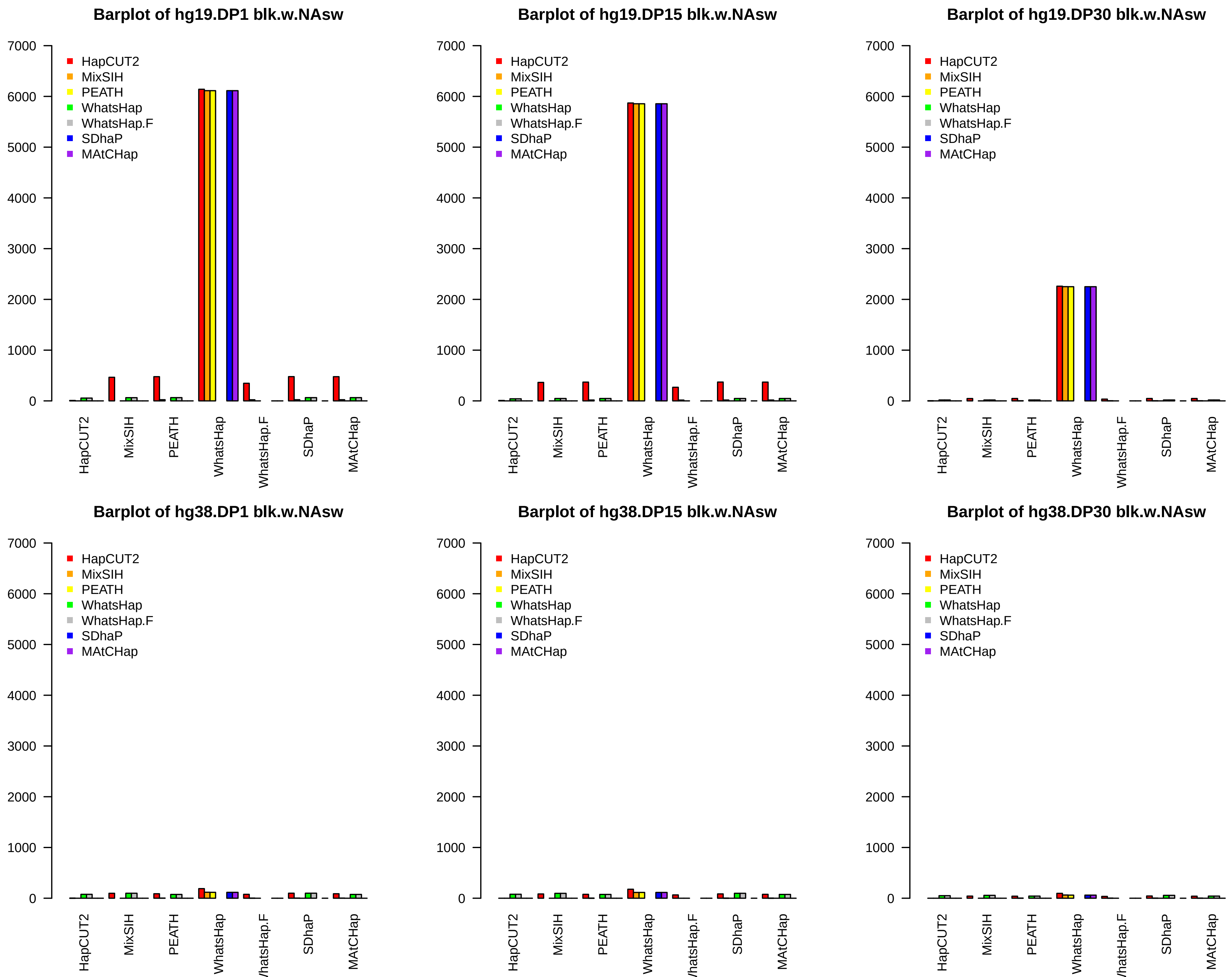
Barplot of hg19.DP1 blk.w.NAsw
Barplot of hg19.DP15 blk.w.NAsw
Barplot of hg19.DP30 blk.w.NAsw
Barplot of hg38.DP1 blk.w.NAsw
Barplot of hg38.DP15 blk.w.NAsw
Barplot of hg38.DP30 blk.w.NAsw
0
1000
2000
3000
4000
5000
6000
7000
HapCUT2
MixSIH
PEATH
WhatsHap
WhatsHap.F
SDhaP
MAtCHap

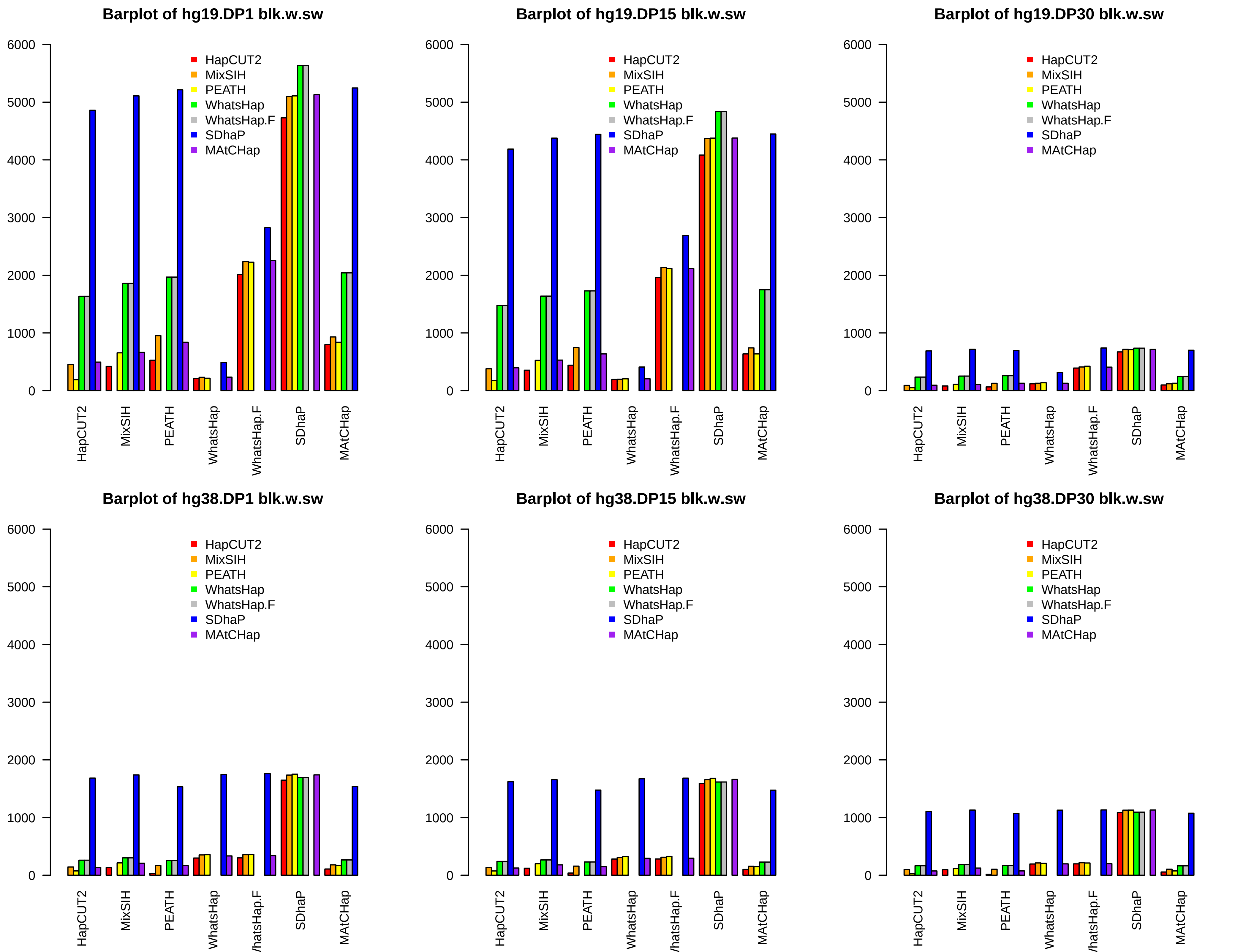
Barplot of hg19.DP1 blk.w.sw
Barplot of hg19.DP15 blk.w.sw
Barplot of hg19.DP30 blk.w.sw
Barplot of hg38.DP1 blk.w.sw
Barplot of hg38.DP15 blk.w.sw
Barplot of hg38.DP30 blk.w.sw
0
1000
2000
3000
4000
5000
6000
HapCUT2
MixSIH
PEATH
WhatsHap
WhatsHap.F
SDhaP
MAtCHap
HapCUT2
MixSIH
PEATH
WhatsHap
WhatsHap.F
SDhaP
MAtCHap
/hatsHap.F

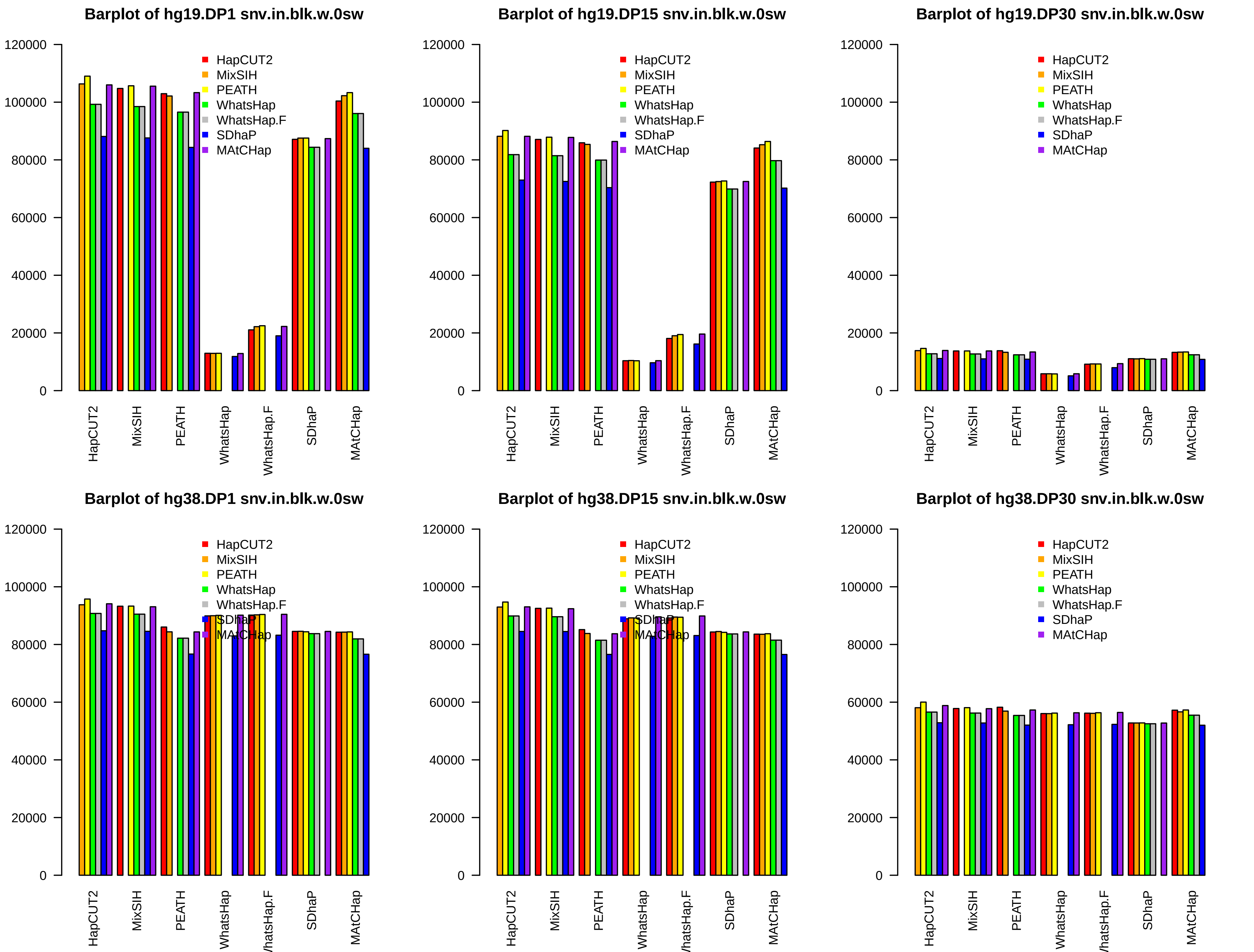

Barplot of hg19.DP1 snv.in.blk.w.0sw
Barplot of hg19.DP15 snv.in.blk.w.0sw
Barplot of hg19.DP30 snv.in.blk.w.0sw
Barplot of hg38.DP1 snv.in.blk.w.0sw
Barplot of hg38.DP15 snv.in.blk.w.0sw
Barplot of hg38.DP30 snv.in.blk.w.0sw
0
20000
40000
60000
80000
100000
120000
HapCUT2
MixSIH
PEATH
WhatsHap
WhatsHap.F
SDhaP
MAtCHap

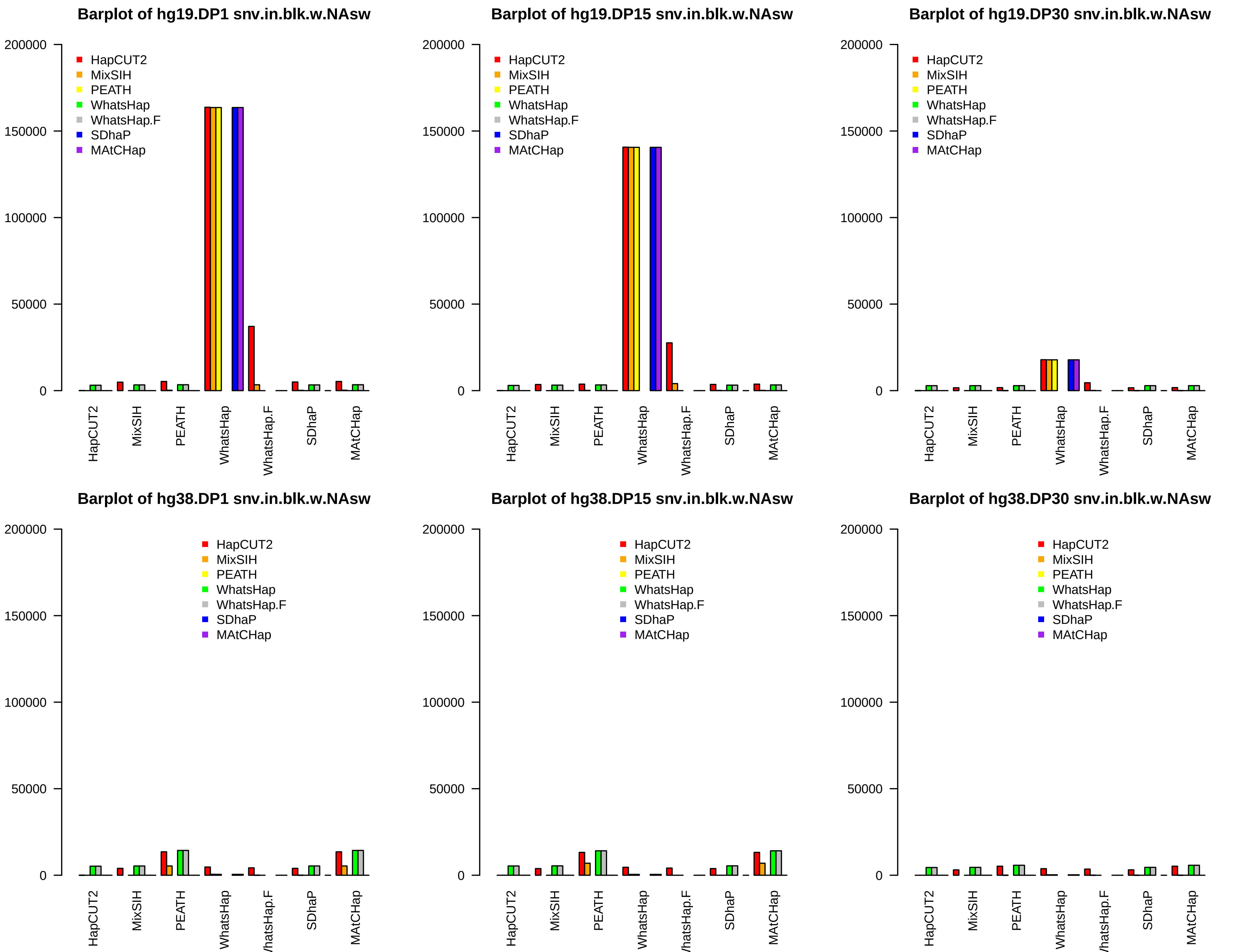
Barplot of hg19.DP1 snv.in.blk.w.NAsw
Barplot of hg19.DP15 snv.in.blk.w.NAsw
Barplot of hg19.DP30 snv.in.blk.w.NAsw
Barplot of hg38.DP1 snv.in.blk.w.NAsw
Barplot of hg38.DP15 snv.in.blk.w.NAsw
Barplot of hg38.DP30 snv.in.blk.w.NAsw
200000
150000
100000
50000
0
HapCUT2
MixSIH
PEATH
WhatsHap
WhatsHap.F
SDhaP
MAtCHap

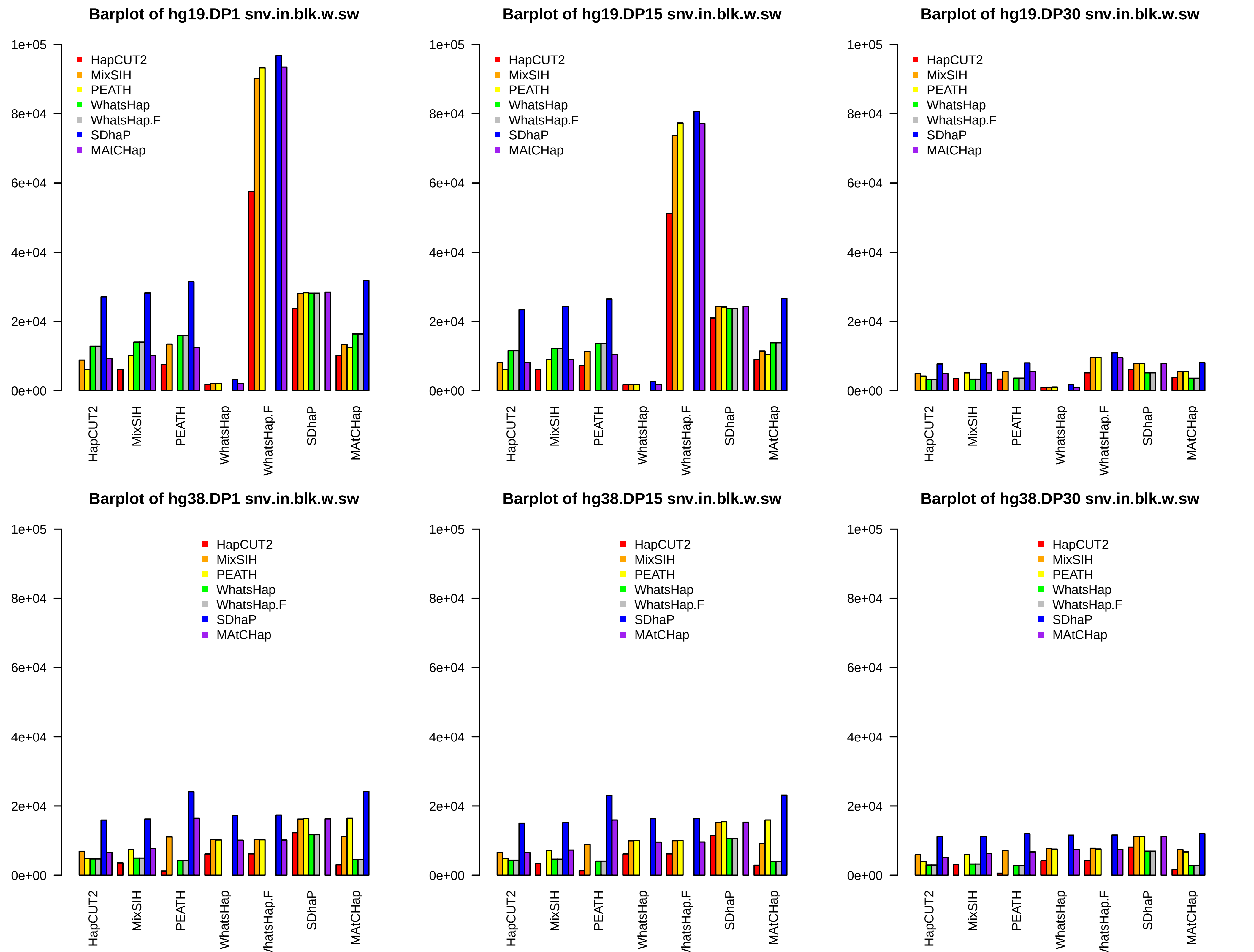
Barplot of hg19.DP1 snv.in.blk.w.sw
Barplot of hg19.DP15 snv.in.blk.w.sw
Barplot of hg19.DP30 snv.in.blk.w.sw
Barplot of hg38.DP1 snv.in.blk.w.sw
Barplot of hg38.DP15 snv.in.blk.w.sw
Barplot of hg38.DP30 snv.in.blk.w.sw
1e+05
8e+04
6e+04
4e+04
2e+04
0e+00
HapCUT2
MixSIH
PEATH
WhatsHap
WhatsHap.F
SDhaP
MAtCHap

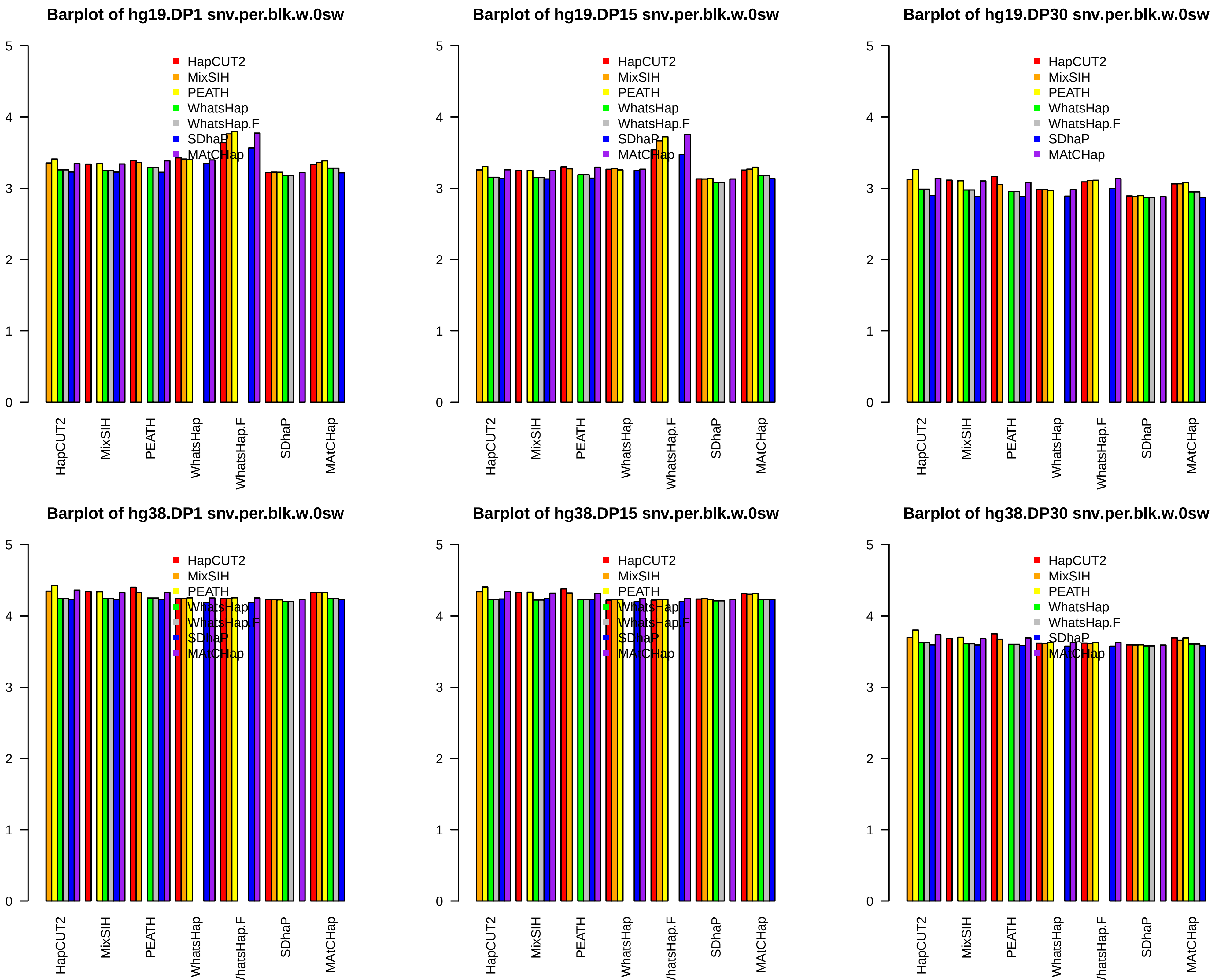
Barplot of hg19.DP1 snv.per.blk.w.0sw
Barplot of hg19.DP15 snv.per.blk.w.0sw
Barplot of hg19.DP30 snv.per.blk.w.0sw
Barplot of hg38.DP1 snv.per.blk.w.0sw
Barplot of hg38.DP15 snv.per.blk.w.0sw
Barplot of hg38.DP30 snv.per.blk.w.0sw
HapCUT2
MixSIH
PEATH
WhatsHap
WhatsHap.F
SDhaP
MAtCHap
0
1
2
3
4
5

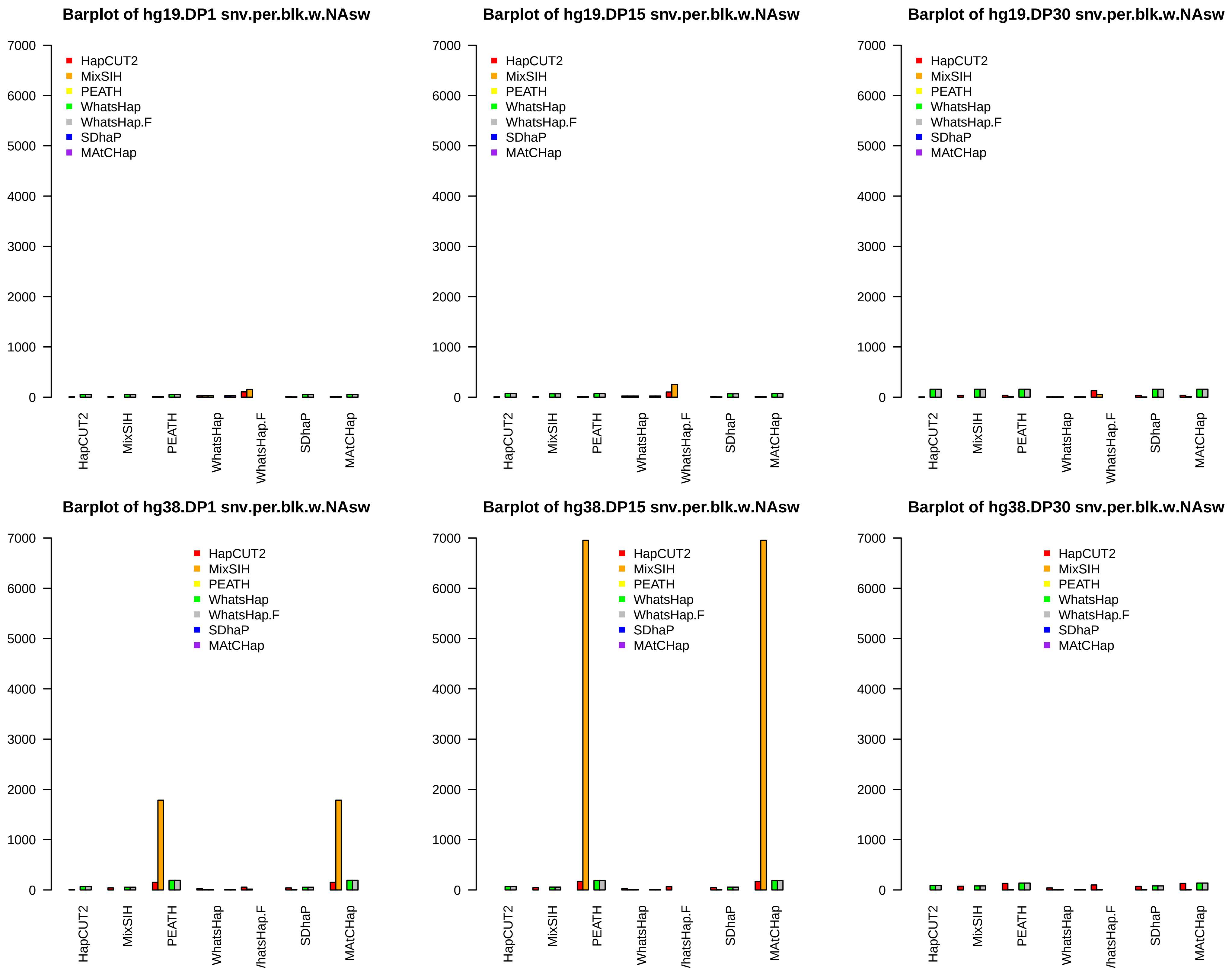

Barplot of hg19.DP1 snv.per.blk.w.NAsw
Barplot of hg19.DP15 snv.per.blk.w.NAsw
Barplot of hg19.DP30 snv.per.blk.w.NAsw
Barplot of hg38.DP1 snv.per.blk.w.NAsw
Barplot of hg38.DP15 snv.per.blk.w.NAsw
Barplot of hg38.DP30 snv.per.blk.w.NAsw
0
1000
2000
3000
4000
5000
6000
7000
HapCUT2
MixSIH
PEATH
WhatsHap
WhatsHap.F
SDhaP
MAtCHap
/hatsHap.F

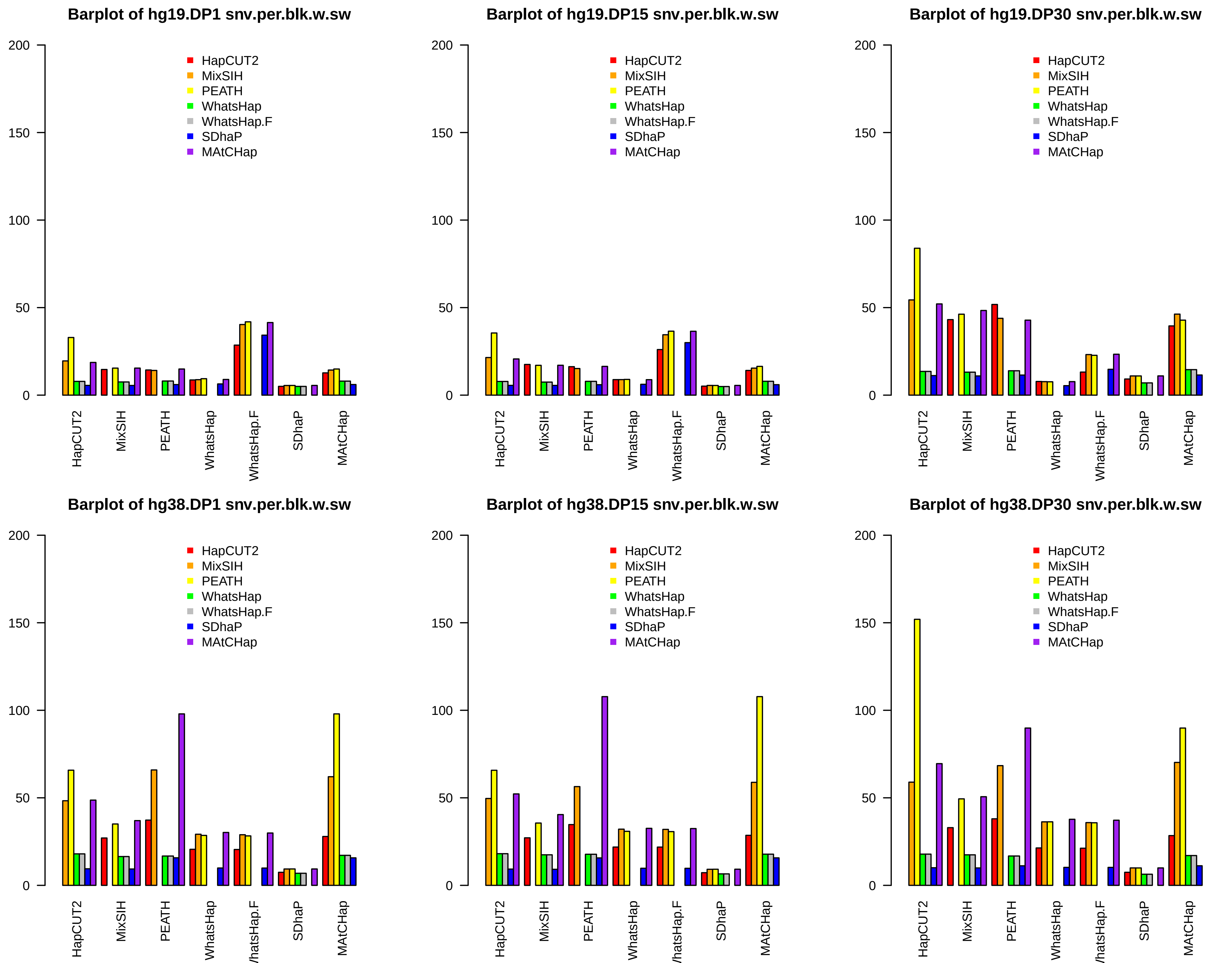
Barplot of hg19.DP1 snv.per.blk.w.sw
Barplot of hg19.DP15 snv.per.blk.w.sw
Barplot of hg19.DP30 snv.per.blk.w.sw
Barplot of hg38.DP1 snv.per.blk.w.sw
Barplot of hg38.DP15 snv.per.blk.w.sw
Barplot of hg38.DP30 snv.per.blk.w.sw
HapCUT2
MixSIH
PEATH
WhatsHap
WhatsHap.F
SDhaP
MAtCHap
0
50
100
150
200

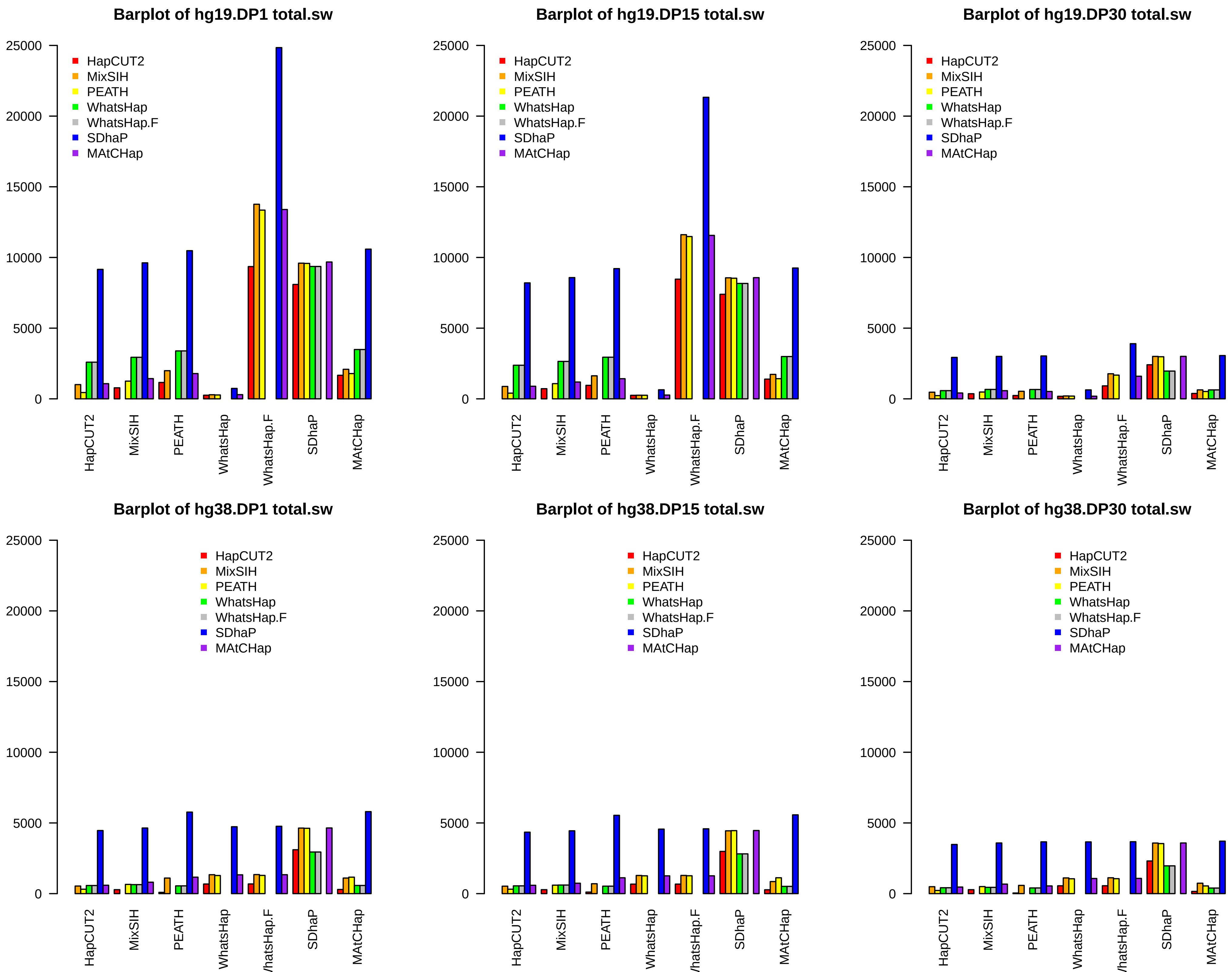
Barplot of hg19.DP1 total.sw
Barplot of hg19.DP15 total.sw
Barplot of hg19.DP30 total.sw
Barplot of hg38.DP1 total.sw
Barplot of hg38.DP15 total.sw
Barplot of hg38.DP30 total.sw
0
5000
10000
15000
20000
25000
HapCUT2
MixSIH
PEATH
WhatsHap
WhatsHap.F
SDhaP
MAtCHap

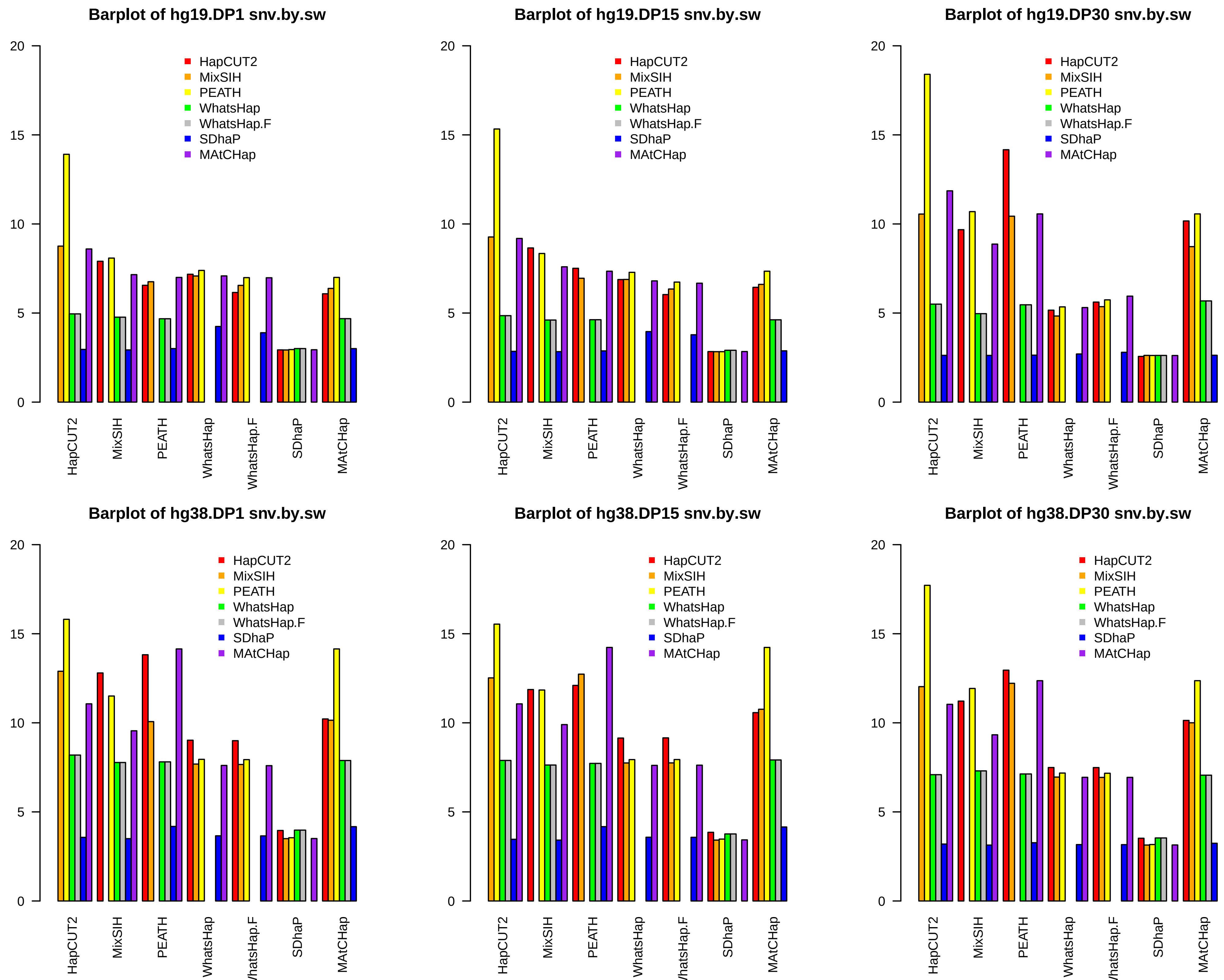
Barplot of hg19.DP1 snv.by.sw
Barplot of hg19.DP15 snv.by.sw
Barplot of hg19.DP30 snv.by.sw
Barplot of hg38.DP1 snv.by.sw
Barplot of hg38.DP15 snv.by.sw
Barplot of hg38.DP30 snv.by.sw
HapCUT2
MixSIH
PEATH
WhatsHap
WhatsHap.F
SDhaP
MAtCHap
0
5
10
15
20

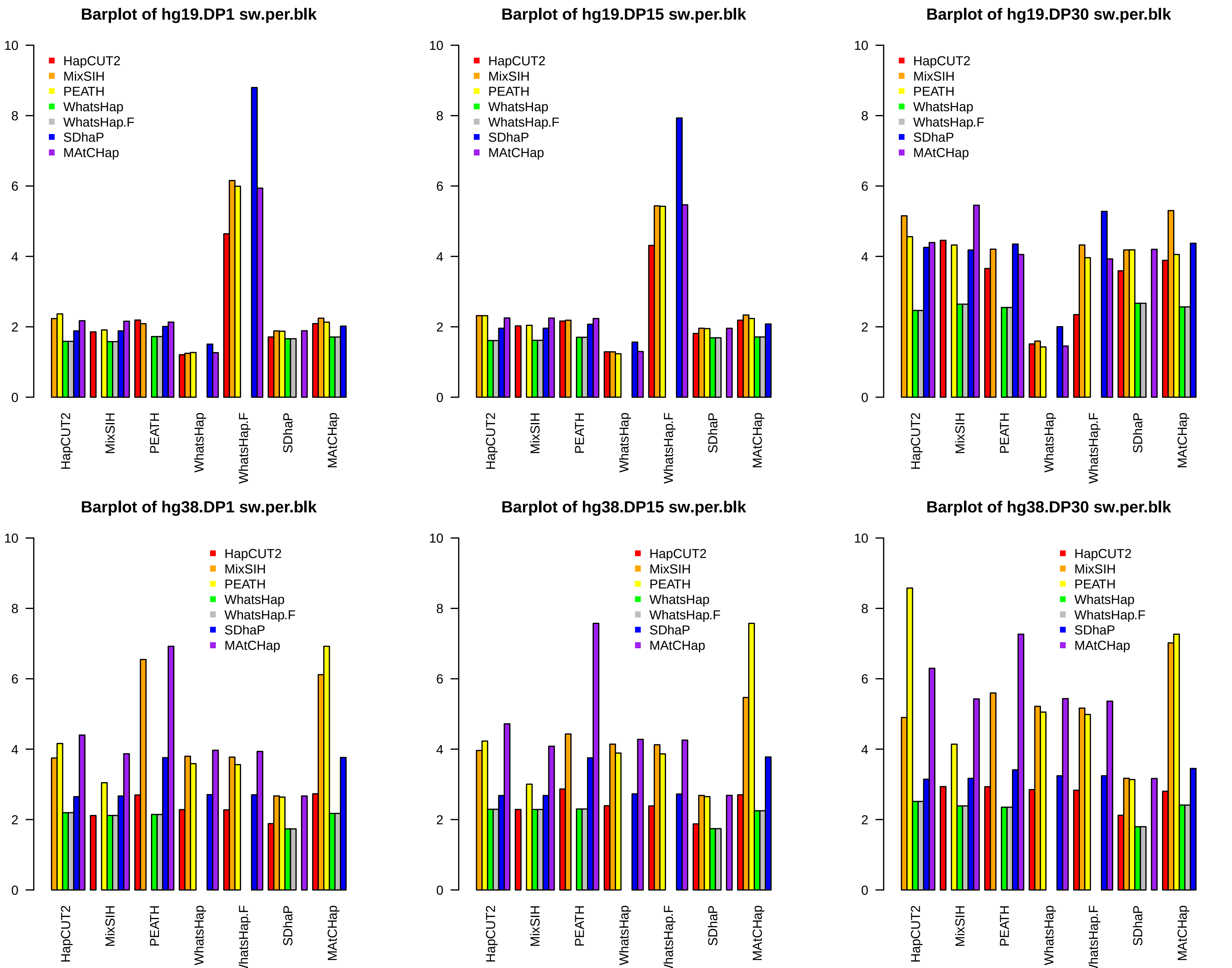
Barplot of hg19.DP1 sw.per.blk
Barplot of hg19.DP15 sw.per.blk
Barplot of hg19.DP30 sw.per.blk
Barplot of hg38.DP1 sw.per.blk
Barplot of hg38.DP15 sw.per.blk
Barplot of hg38.DP30 sw.per.blk
HapCUT2
MixSIH
PEATH
WhatsHap
WhatsHap.F
SDhaP
MAtCHap
0
2
4
6
8
10
